# Cell migration signaling through the EGFR-VAV2-Rac1 pathway is sustained in endosomes

**DOI:** 10.1242/jcs.263541

**Published:** 2025-01-24

**Authors:** Itziar Pinilla-Macua, Sachin Surve, Alexander Sorkin

**Affiliations:** Department of Cell Biology, University of Pittsburgh School of Medicine, Pittsburgh, PA 15261, USA

**Keywords:** EGFR, Endocytosis, VAV2, Rac1, Cell motility

## Abstract

Ligand binding to EGFR activates Rho family GTPases, triggering actin cytoskeleton reorganization, cell migration and invasion. Activated EGFR is also rapidly endocytosed but the role of EGFR endocytosis in cell motility is poorly understood. Hence, we used live-cell microscopy imaging to demonstrate that endogenous fluorescently labeled VAV2, a guanine nucleotide exchange factor for Rho GTPases, is co-endocytosed with EGFR in genome-edited human oral squamous cell carcinoma (HSC3) cells, an *in vitro* model for head-and-neck cancer where VAV2 is known to promote metastasis and is associated with poor prognosis. Chemotactic migration of HSC3 cells toward an EGF gradient is found to require both VAV2 and clathrin-mediated endocytosis. Moreover, sustained activation of Rac1, a Rho family GTPase promoting cell migration and a major substrate of VAV2, also depends on clathrin. Endogenous fluorescently labeled Rac1 localizes to EGFR-containing endosomes. Altogether, our findings suggest that signaling through the EGFR-VAV2-Rac1 pathway persists in endosomes and that this endosomal signaling is required for EGFR-driven cell migration.

## Teaser

**Highlighted Article:** Cell motility has a major role in development, tissue homeostasis and tumor metastatic processes. EGFR promotes migration of squamous cell carcinoma cells by activating the VAV2-Rac1 signaling pathway in endosomes.

## INTRODUCTION

EGFR is a classical receptor tyrosine kinase (RTK) that is essential during eucaryotic development and for tissue homeostasis (Sibilia et al., 2007). EGFR overexpression or mutations resulting in dysregulation of receptor signaling are found in various types of cancer, thus making EGFR a major therapeutic target and prognostic marker (Uribe et al., 2021). Ligand binding to the receptor at the cell surface initiates a multitude of signaling processes leading to cell division, differentiation, motility and metabolic changes (Lemmon et al., 2014). At the same time, ligand-bound receptors are internalized into endosomes and efficiently sorted to lysosomes for degradation (Goh and Sorkin, 2013). EGFR maintains its activity in endosomes (Lai et al., 1989). However, whether EGFR complexes continue their downstream signaling from endosomes remains under debate (von Zastrow and Sorkin, 2021). A significant gap in understanding the spatiotemporal regulation of EGFR and other RTK signaling is attributable to the limited information about the subcellular localization dynamics of endogenous signaling proteins downstream from activated EGFR. An example of studying the EGFR-Ras-Raf-ERK1/2 (ERK1 and ERK2 are also known as MAPK3 and MAPK1, respectively) pathway illustrates the importance of such information. Whereas the detection of overexpressed Ras and Raf in endosomes supports the hypothesis of signaling from endosomes, studies of endogenous Ras and Raf in living cells have failed to detect these proteins in EGFR-containing endosomes and therefore there are questions regarding this hypothesis (Pinilla-Macua et al., 2016; Surve et al., 2021, 2019). By contrast, time-resolved analysis of the EGFR proximity proteome suggests that several signaling effectors remain associated or proximal to internalized EGFR (Perez Verdaguer et al., 2022).The time-course of EGFR proximity abundance of VAV2, a guanine nucleotide exchange factor (GEF) for Rho family small GTPases, was similar to that of clathrin light chain and the early endosome protein EEA1, suggesting that VAV2 might remain in the proximity of EGFR during clathrin-mediated endocytosis (CME) and in endosomes (Perez Verdaguer et al., 2022).

VAV2, a member of the VAV family of GEFs, is involved in cell growth, differentiation and migration through the regulation of signaling pathways leading to actin cytoskeleton re-organization, membrane ruffling, lamellipodia and invadopodium formation (reviewed in Rodriguez-Fdez and Bustelo, 2019). VAV2 is expressed in a wide range of tissues, including brain, heart and lungs, and in the immune system. The deregulation of VAV2 expression or activity has been associated with cancer, autoimmune and neurological disorders (Huang et al., 2019; Patel et al., 2007; Pickering et al., 2021; Rodriguez-Fdez et al., 2021; Ruggiero and Lalli, 2017). VAV2 overexpression has been observed in breast, colon, lung and other cancers, where it promotes cell proliferation, invasion and metastasis (Rodriguez-Fdez and Bustelo, 2019). In head-and-neck carcinoma, VAV2 overexpression correlates with poor prognosis (Lorenzo-Martin et al., 2020).

VAV2 molecules contain a Dbl Homology (DH) domain, which has GEF activity towards Rho family GTPases, a pleckstrin homology domain, which binds to phosphatidylinositol lipids, an SH2 domain, which mediates interactions with phosphotyrosine-containing motifs, and SH3 domains, which interact with proline-rich motifs. The activation of VAV2 by EGFR and other signaling receptors leads to GTP loading of Rho family GTPases, such as Rac1, Cdc42 and RhoA, which regulate actin cytoskeleton rearrangements, cell adhesion and motility (Abe et al., 2000; Liu and Burridge, 2000). The SH2 domain is thought to directly interact with activated EGFR resulting in tyrosine phosphorylation of VAV2 and conformational changes necessary for the DH domain activity (Lopez-Lago et al., 2000; Tamas et al., 2003). However, the localization of endogenous VAV2 has not been studied in living cells, and whether VAV2-mediated signaling from EGFR to actin cytoskeleton is regulated by EGFR endocytosis is unknown. Hence, given the importance of VAV2 in head-and-neck cancer, we used human oral squamous cell carcinoma cell line HSC3 to fluorescently label endogenous VAV2 and its major substrate, Rac1, by CRISPR/Cas9 gene-editing. Live-cell fluorescence microscopy imaging demonstrated the presence of VAV2 and Rac1 in EGFR-containing endosomes. EGF-induced Rac1 activation and EGF-guided chemotactic migration of HSC3 cells were both VAV2 and clathrin dependent. Therefore, we propose that the EGFR-VAV2-Rac1 signaling axis remains operational in endosomes, and that this endosomal signaling is important for EGF-induced cell motility.

## RESULTS

**Fig. 1.**
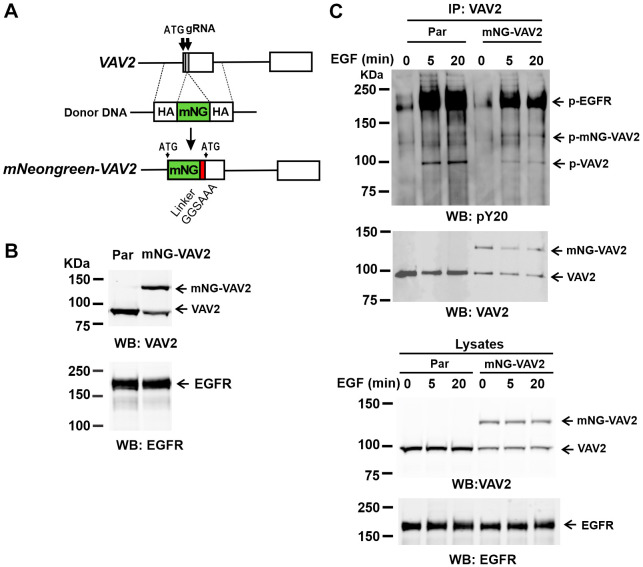
**Labeling of endogenous VAV2 with mNG in HSC3 cells and its EGF-dependent phosphorylation**. (A) Schematics of the insertion of the mNG sequence into the endogenous locus in *VAV2* gene. HA, homology arm. (B) Lysates of parental (par) HSC3 and pooled HSC3 mNG-VAV2 cells were probed by western blotting (WB) with anti-VAV2 and -EGFR (loading control) antibodies. (C) Parental HSC3 cells and HSC3 mNG-VAV2 cells were serum starved and treated with 4 ng/ml EGF for indicated times at 37°C. Cells were lysed, and VAV2 was immunoprecipitated (IP). Immunoprecipitates and aliquots of lysates (20%) were electrophoresed and probed by western blotting with antibodies to phosphotyrosine (pY20), EGFR and VAV2. Blots in B and C representative of two to four repeats.

To elucidate the role of endocytosis in EGFR signaling to cell motility through VAV2, we examined the localization of endogenous VAV2 in EGF-stimulated HSC3 cells. The rationale for using HSC3 cells was two-fold: these cells (1) have growth that is dependent on EGFR activity (Ohnishi et al., 2008; Pinilla-Macua et al., 2017), and (2) represent an experimental model of the squamous cell carcinoma of head-and-neck, a tumor type in which VAV2 is an emerging prognostic marker (Lorenzo-Martin et al., 2020). To generate endogenously labeled VAV2, mNeonGreen (mNG) sequence was inserted at the 5′-end of *VAV2* gene using CRISPR/Cas9 gene editing (Fig. 1A). Because HSC3 cells do not survive single-cell cloning, a pool of cells (hereafter termed ‘mNG–VAV2’ cells) was generated in which both untagged (∼100 kDa) and mNG-tagged VAV2 (∼125 kDa) were detected in comparable amounts (Fig. 1B). This indicates that most cells carry one edited *VAV2* gene allele. Previous studies using recombinant VAV2 have demonstrated full functionality of VAV2 tagged at the N-terminus, including its activation by EGFR-mediated tyrosine phosphorylation (Lopez-Lago et al., 2000; Tamas et al., 2003). Immunoblotting of VAV2 immunoprecipitates from parental and mNG–VAV2 HSC3 cells revealed a similar level of tyrosine phosphorylation in untagged and mNG-tagged VAV2 in EGF-treated cells, indicative of a normal activation of mNG–VAV2 (Fig. 1C). Moreover, the amount of phosphorylated EGFR detected in VAV2 immunoprecipitates was proportional to the amount of immunoprecipitated VAV2 and mNG–VAV2, indicative of a comparable association of untagged VAV2 and mNG–VAV2 with activated EGFR. These experiments validated the use of mNG–VAV2 cells for studying VAV2 localization dynamics.

**Fig. 2.**
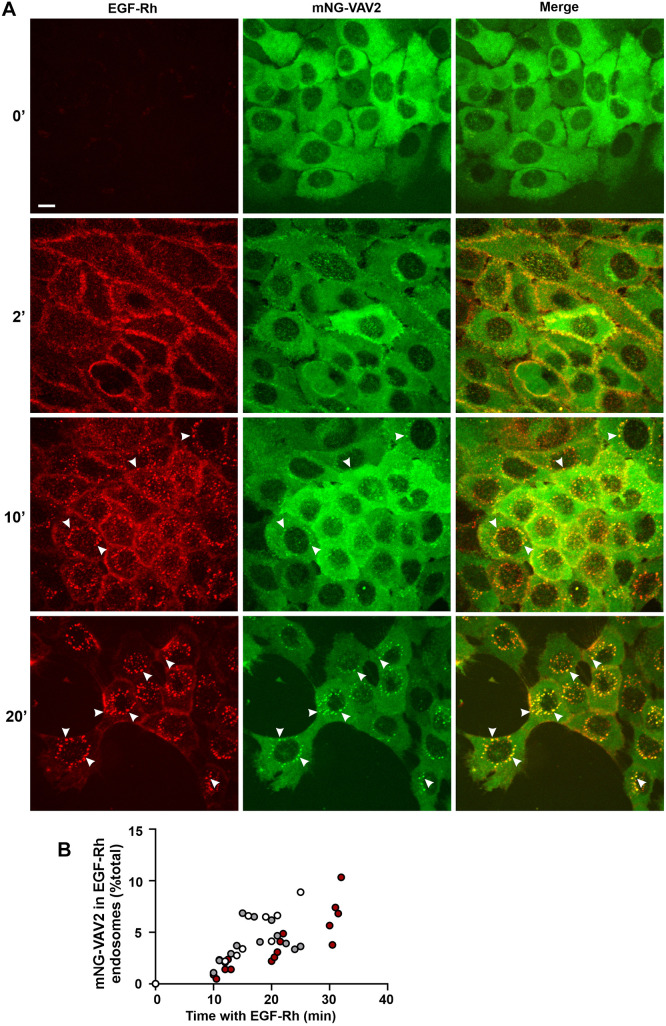
**Localization dynamics of endogenous mNG–VAV2 during cell stimulation with EGF–Rh.** (A) HSC3 mNG–VAV2 cells were serum starved and incubated with 4 ng/ml EGF–Rh at 37°C for the indicated times. 3D live-cell imaging was performed through 488-nm (green; mNG–VAV2), 561-nm (red; EGF–Rh) and 640-nm (autofluorescence, see Fig. S1A) channels. Fluorescence intensity scales are identical at all time points. Individual confocal sections are shown. Examples of colocalization of mNG–VAV2 and EGF–Rh in endosomes are indicated by arrowheads. ', minute. Scale bar: 10 μm. (B) Quantification of colocalization of mNG–VAV2 with EGF–Rh in endosomes. The fractions of total mNG–VAV2 colocalized with EGF–Rh were calculated from images as shown in A in three independent time-course experiments (indicated by different colored symbols). Each data point represents a single field of view (FOV).

Studies in mouse tumor xenografts of HSC3 cells have demonstrated that EGFR ligands are present in picomolar concentrations (<1–2 ng/ml) *in vivo* and that these concentrations are sufficient for tumor progression (Ohnishi et al., 2008; Pinilla-Macua et al., 2017). However, using such low EGF concentrations for single-cell microscopy analysis of the localization dynamics of endogenously labeled proteins, such as mNG–VAV2, poses technical challenges, mainly due to substantial autofluorescence from endolysosomal compartments, which results in a low signal-to-noise ratio of the mNG fluorescence. Therefore, we used a near-physiological concentration of EGF–Rhodamine conjugate (EGF–Rh) (4 ng/ml) to monitor the subcellular localization of activated EGFR and mNG–VAV2 by live-cell three-dimensional (3D) imaging. Before stimulation, mNG–VAV2 was diffusely distributed in the cytosol, although accumulations of apparent mNG fluorescence were sporadically seen in some cells (Fig. 2A). Such accumulations, however, displayed a wide-spectrum fluorescence characteristic of endolysosomal autofluorescence, which was monitored by imaging the 640-nm channel fluorescence as described previously (Surve et al., 2019). Within 1–3 min after EGF–Rh stimulation, recruitment of mNG–VAV2 to the plasma membrane including protrusions, ruffles and lamellipodia was observed, where mNG–VAV2 was colocalized with EGF–Rh. At later times, mNG–VAV2 was detected in endosomes containing EGF–Rh with the maximal extent of colocalization after 20–30 min of EGF–Rh stimulation (Fig. 2A,B; Movie 1). EGF–Rh:mNG–VAV2-containing endosomes showed a minimal overlap with the 640-nm channel fluorescence (autofluorescence) (Fig. S1A). The fluorescence intensity through the 488-nm channel of EGF–Rh-containing endosomes in mNG–VAV2 cells was an order of magnitude stronger than the 488-nm channel autofluorescence in endosomes of parental HSC3 cells stimulated with EGF–Rh (Fig. S1B,C), thus confirming the specificity of the endosomal mNG–VAV2 fluorescence. On average, ∼3–7% of total cellular mNG–VAV2 was located in EGFR-containing endosomes (Fig. 2B), whereas a large amount of mNG–VAV2 remained in the cytosol throughout the 30-min time-course. Such inefficient VAV2:EGFR association can be explained by the fact that in cells stimulated with 4 ng/ml EGF, only ∼5000 EGFR molecules per cell are initially activated. Therefore, multiple SH2 or phosphotyrosine-binding (PTB) domain-containing proteins, such as Grb2, PLCγ1, Cbl proteins, the p85 phosphoinositide 3-kinase (PI3K) subunit, Shc, Nck and Crk, compete with VAV2 for binding to a few activated receptors. Because several of the above-mentioned proteins are more abundant than VAV2, they might outcompete VAV2 for binding to active EGFR. In support of the latter point, an ∼7-fold larger fraction of mNG–VAV2 was detected in EGFR-containing endosomes of cells stimulated with 40 ng/ml EGF–Rh than in cells stimulated with 4 ng/ml EGF–Rh (Fig. S2).

Altogether, the data presented in Fig. 2 demonstrate, for the first time, endosomal localization of endogenous VAV2 in living cells stimulated with low concentrations of EGF, thus implying that VAV2-mediated signaling from EGFR to Rho GTPases and cell motility can be sustained in endosomes under physiological conditions. Previously, overexpressed recombinant VAV2 was detected in endosomes of EGF-stimulated HeLa cells (Thalappilly et al., 2010), although a very high EGF concentration, VAV2 overexpression and cell fixation were used in the latter study, which could have resulted in mistargeting of VAV2 and an overestimation of the amount of VAV2 associated with EGFR in endosomes.

**Fig. 3.**
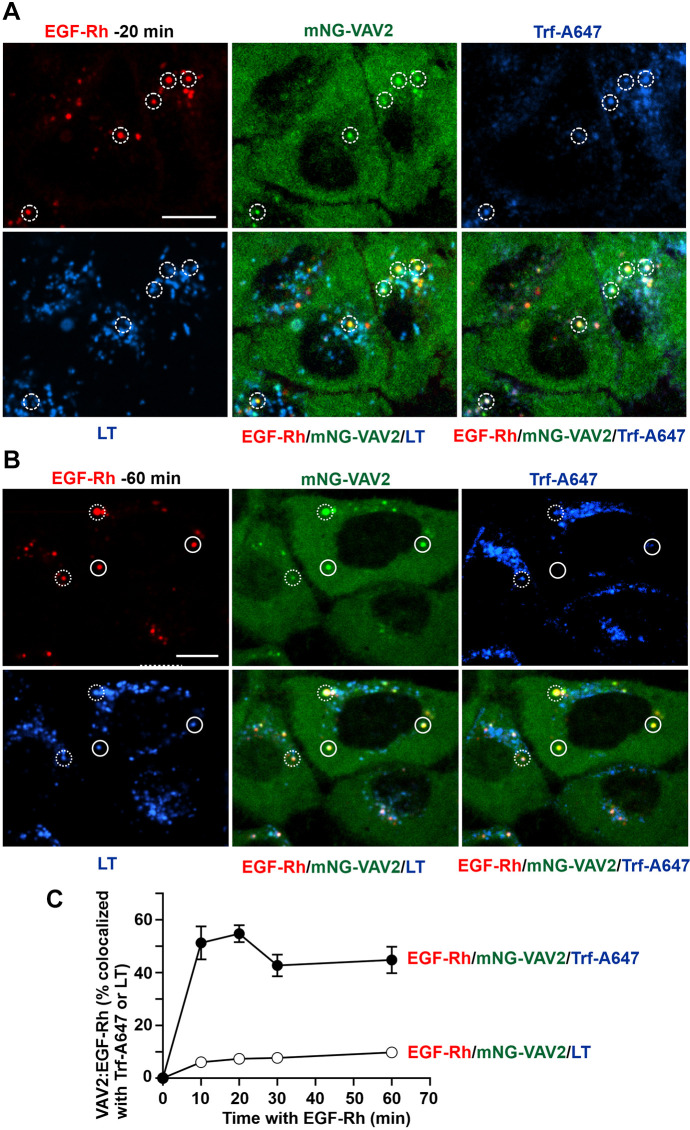
**Internalized EGF–Rh and mNG–VAV2 are predominantly located in early, sorting and recycling endosomes during the first hour of continuous endocytosis.** mNG–VAV2 cells were serum starved and pretreated with Trf–A647 and LysotrackerBlue (LT) for 5 min. The cells were then incubated with EGF–Rh (4 ng/ml) and Trf–A647 (5 µg/ml) at 37°C for 20 min (A) or 60 min (B). 3D live-cell imaging was performed through 405-nm (blue, LT), 488-nm (green, mNG-VAV2), 561-nm (red, EGF–Rh) and 640-nm (blue, Trf–A647) channels. Dotted circles indicate examples of EGF–Rh, mNG–VAV2 and Trf–A647 colocalization (without or with LT, presumably, early and sorting endosomes, respectively), whereas unbroken circles show colocalization of EGF–Rh, mNG–VAV2 and LT but not with Tfr–A647 (presumably, late endosomes and lysosomes). Scale bars: 10 µm. (C) Quantification of the fraction of mNG–VAV2 colocalized with EGF–Rh and Trf–A647 (without and with LT; ‘early, sorting and recycling endosomes’) or LT (late endosomes and lysosomes, no Trf–A647) from images as shown in A and B. The data points represent mean±s.e.m. from 5–6 FOVs at each time point. Error bars are not shown if they are smaller than symbols. This experiment is representative of three independent time-course experiments.

To characterize the nature of EGF–Rh-containing endosomes in mNG–VAV2 HSC3 cells by live-cell microscopy with minimal perturbations, the cells were prelabeled with LysoTrackerBlue (to label late endosomes and lysosomes) and stimulated with EGF–Rh in the presence of transferrin conjugated with Alexa Fluor 647 (Trf–A647; a marker of early, sorting and recycling endosomes). Live-cell imaging revealed that EGF–Rh and mNG–VAV2 were initially (first 20–30 min) colocalized with vesicular compartments containing Trf–A647 (Fig. 3A,C), and that this level of colocalization was sustained during the first hour of EGF–Rh stimulation (Fig. 3B,C). A fraction of sorting endosomes, containing both Trf–A647 and LysoTrackerBlue, were positive for EGF–Rh and mNG–VAV2 during the first hour of EGF–Rh stimulation (Fig. 3A,B). EGFR and mNG–VAV2 accumulated in late endosomes and lysosomes (positive for LysoTrackerBlue but lacking Trf–A647) at a significantly slower rate and in smaller amounts compared to in early and sorting endosomes (Fig. 3C). Such a relatively slow maturation process for EGFR-containing endosomes is consistent with the slow degradation of EGFR stimulated by low EGF concentrations in HSC3 cells (Pinilla-Macua et al., 2017). Overall, these data indicate that mNG–VAV2 is associated with EGFR mainly in early and sorting endosomes during the first hour of continuous receptor endocytosis in HSC3 cells.

### EGF-guided chemotactic cell migration and substrate spreading require VAV2

**Fig. 4.**
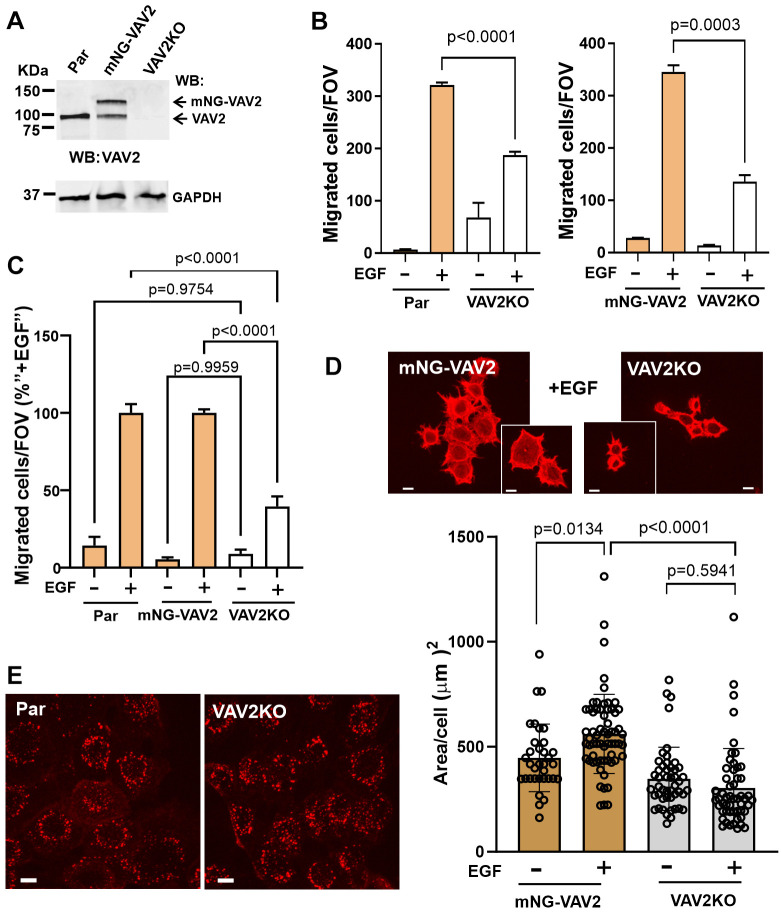
**VAV2 is necessary for EGF-directed chemotactic migration and EGF-dependent substrate spreading of HSC3 cells but not for EGFR endocytosis.** (A) Lysates of parental HSC3 (Par), mNG–VAV2 and VAV2KO cells were resolved by western blotting (WB) and probed with anti-VAV2 and -GAPDH (loading control) antibodies. Blots representative of three repeats. (B) Parental HSC3 and VAV2KO cells (left) or mNG–VAV2 and VAV2KO cells (right) were plated in the upper chamber of pre-treated Transwell inserts (10^5^ cells/insert). Medium with or without 4 ng/ml EGF was added to the bottom compartment of wells. Cells were incubated for 4 h at 37°C to allow cell migration to the bottom surface of Transwell inserts, fixed and stained with Crystal Violet. Cells that had migrated to the bottom surface of Transwell inserts were imaged. Bar graphs represent mean±s.e.m. numbers of cells per FOV (6 FOVs per insert; *n*=3 inserts). (C) Combined analysis of multiple migration experiments as per B. Bar graph represents mean±s.e.m. numbers of migrated cells per FOV in each individual Transwell insert normalized to the mean number of migrated cells per FOV in ‘+EGF’ inserts in each independent experiment (total *n*=6–13 inserts per condition). *P*-values in B and C were calculated using one-way ANOVA followed by a Tukey's multiple comparison test. (D) mNG–VAV2 and VAV2KO cells were plated onto coverslips for 1 h, fixed and stained with phalloidin–TxR. A *z*-stack of images was acquired through the 561-nm laser channel. Examples of representative maximum intensity projection images are shown. Scale bars: 10 µm. Quantifications of the total area of individual cell footsteps were performed using maximum intensity projection images as described in the Materials and Methods and are shown in the graph below images. Mean±s.d. are shown. Each data point represents an individual cell. This experiment is representative of two independent experiments. *P* values were calculated using one-way ANOVA followed by a Tukey's multiple comparison test. (E) Parental HSC3 and VAV2KO cells were incubated with 4 ng/ml EGF–Rh at 37°C for 15 min, and live-cell imaging was performed. Maximum fluorescence intensity projections (MIPs) of *z*-stack of confocal images are shown. Images are representative of five to ten repeats. Scale bars: 10 µm.

To examine the role of endosomal localization of VAV2 in EGFR signaling, we first tested whether VAV2 is required for HSC3 cell motility. To this end, we generated a VAV2 knockout in mNG–VAV2 cells (hereafter termed ‘VAV2KO cells’) using CRISPR/Cas9 gene-editing (Fig. 4A). Previous studies demonstrated that VAV2 can be involved in EGF-guided chemotactic cell migration and in spreading of cells on the substrate (Duan et al., 2011; Marignani and Carpenter, 2001; Patel et al., 2007). However, VAV2 is one of at least three GEFs for Rac1, and elimination of VAV2 does not affect RTK-induced cell motility and cell spreading in several cell types because of this functional redundancy (Itoh et al., 2008; Marignani and Carpenter, 2001). To test the effects of VAV2 knockout on EGF-induced cell migration of HSC3 cells, a chemotactic migration assay using a Boyden chamber was employed. In these experiments, migration of cells through pores of Transwell inserts towards medium containing 4 ng/ml EGF was monitored. As shown in Fig. 4B,C, migration of both parental HSC3 and mNG–VAV2 cells was EGF dependent. Elimination of VAV2 significantly reduced the number of cells migrated towards EGF gradient but did not significantly affect EGF-independent migration (Fig. 4B,C), thus demonstrating the essential role of VAV2 in EGFR-induced signaling processes leading to invasive migration of HSC3 cells. Interestingly, cell migration was not promoted by 40 ng/ml EGF or 10% fetal bovine serum (FBS) (Fig. S3). This outcome was likely due to a substantial downregulation of EGFR during the 4-h motility assay (Pinilla-Macua et al., 2017) and the low concentrations of EGFR ligands in FBS. RNAi-mediated depletion of VAV2 has been previously shown to decrease the migration of MDA-MD-468 cells towards gradient of hepatocyte growth factor (HGF), a ligand for the c-MET RTK (Menard et al., 2014; Palamidessi et al., 2008). HGF also stimulated HSC3 cell migration in a VAV2-dependent manner in our experiments (Fig. S3), indicative of the common signaling processes downstream of EGFR and c-MET. Furthermore, we found that EGF (4 ng/ml) accelerates the spreading of HSC3 cells on the substrate. In these experiments, areas of cell footprints were measured 1 h after plating cells at low density (Fig. 4D). VAV2 knockout diminished EGF-dependent cell spreading (Fig. 4D).

VAV2 has been proposed to be involved in EGFR trafficking in HeLa cells by slowing down EGF-induced EGFR endocytosis (Thalappilly et al., 2010), although these results were obtained using an exceedingly high EGF concentration, which favors EGFR internalization via the clathrin-independent pathway in HeLa cells (Sigismund et al., 2005). VAV2 knockout did not affect endocytosis of 4 ng/ml EGF–Rh in HSC3 cells, suggesting that inhibition of EGFR-dependent cell migration and substrate-spreading by VAV2 knockout were not due to impaired CME of EGFR (Fig. 4E). Together, the data in Fig. 4 and Fig. S3 demonstrate that HSC3 cells are a valid model to study the role of endocytosis in the EGFR-VAV2 signaling axis, which promotes cell motility.

### EGF-guided chemotactic cell migration requires clathrin-mediated endocytosis

**Fig. 5.**
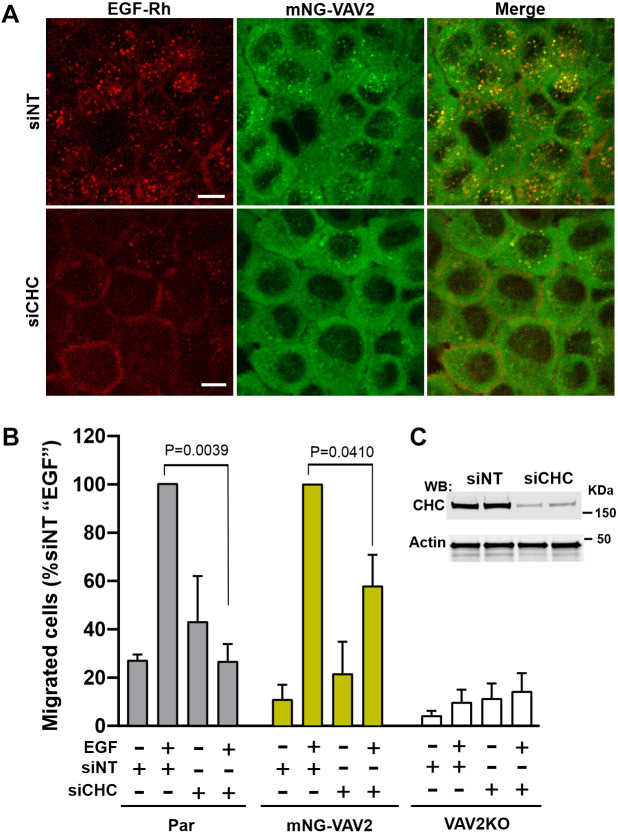
**Inhibition of CME decreases EGF-guided migration of cells.** (A) mNG–VAV2 cells were transfected with non-targeting (siNT) and clathrin heavy chain (CHC)-targeting siRNA (siCHC). Cells were incubated with 4 ng/ml EGF–Rh at 37°C for 15 min, and live-cell imaging was performed. Scale bars: 10 µm. Maximum fluorescence intensity projections (MIPs) of *z*-stack of confocal planes are shown. (B) Parental HSC3, mNG–VAV2 and VAV2KO cells transfected with siNT or siCHC were plated in the upper chamber of pre-treated Transwell inserts (10^5^ cells/insert). Medium with or without 4 ng/ml EGF was added to the bottom compartment of wells. Cells were incubated for 4 h at 37°C to allow cell migration to the bottom surface of Transwell inserts, fixed and stained with Crystal Violet. Cell migrated to the bottom surface of Transwell inserts were imaged and counted. Bar graphs represent mean±s.e.m. numbers of cells per FOV normalized to the mean number in siNT-transfected cells stimulated with EGF in each individual experiment for mNG–VAV2 and parental HSC3 cells. The number of migrated VAV2KO cells were normalized to the mean number of EGF-treated siNT parental HSC3 cells in each experiment. The data are combined from two or three independent experiments for each cell clone (10–11 FOVs per experimental variant). *P*-values were calculated using one-way ANOVA followed by a Tukey's multiple comparison test. Differences between the numbers of siNT- and siCHC-transfected EGF-untreated cells (parental and mNG-VAV2) were not significant. (C) Example of CHC knockdown efficiency. Lysates of parental HSC3 cells transfected with siNT or siCHC were resolved by SDS-PAGE and probed with CHC and Akt (loading control) antibodies.

To investigate the role of endocytosis in VAV2-mediated signaling by EGFR, clathrin heavy chain (CHC; also known as CLTC) was depleted using a well-characterized siRNA duplex 2 (Huang et al., 2004). CHC knockdown strongly inhibited endocytosis of EGF–Rh (4 ng/ml) in most mNG–VAV2-expressing cells (Fig. 5A). Despite efficient CHC knockdown (85–93%) in most experiments (Fig. 5C), EGFR internalization was still observed in a small subset of cells, presumably, due to residual CHC and/or clathrin-independent endocytosis. Recruitment of mNG–VAV2 to endosomes decreased proportionally with the reduction of EGF–Rh in endosomes (Fig. 5A). Phosphorylation of EGFR at Tyr992 and Tyr1173, sites shown to be important for VAV2 interaction with EGFR (Tamas et al., 2003) was unaffected by CHC knockdown (Fig. S4). This suggests that the lack of mNG–VAV2 in endosomes of CHC-depleted cells was not due to diminished EGFR phosphorylation.

Because EGF and VAV2 dependency were more pronounced in the migration assay than in the cell spreading assay, we measured cell migration in cells depleted of CHC. CHC knockdown significantly reduced the number of parental and mNG–VAV2 HSC3 cells migrating in an EGF-dependent manner, whereas EGF-independent migration was not significantly affected (Fig. 5B). These experiments demonstrate that EGF-guided cell migration requires CHC, suggesting the importance of CME in EGFR signaling to cell migration.

### EGF-dependent Rac1 activation is regulated by endocytosis

**Fig. 6.**
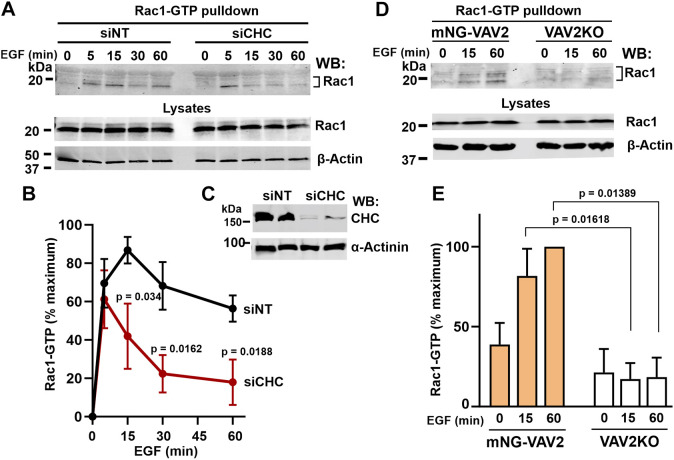
**CME is required for sustained VAV2-dependent Rac1 activation by EGFR.** (A) Parental HSC3 cells were transfected with non-targeting (siNT) and CHC targeting siRNA (siCHC). The cells were stimulated with 10 ng/ml EGF for indicated times, and GTP-loaded Rac1 (Rac1-GTP) was pulled down from cell lysates with GST–PBD. Pulldowns and aliquots of lysates were resolved by SDS-PAGE and probed with Rac1 and β-actin antibodies (loading control). (B) Quantification of the amount of Rac1-GTP pulled down from six independent experiments as shown in A. The amount of Rac1-GTP (pulldowns) was corrected by the amount of β-actin in corresponding lysates. The amount of corrected Rac1-GTP at time ‘0’ was subtracted from these amounts at other time points in each individual time-course experiment. The resulting values are expressed as a percentage of the maximal Rac1-GTP amount in siNT-transfected cells within each time-course experiment. Mean±s.e.m. (*n*=6) is presented. *P*-values for individual time-points were calculated using an unpaired two-tailed Student's *t-*test against siNT-transfected cells. (C) Example of CHC knockdown efficiency in experiments described in A and B. Lysates of parental HSC3 cells transfected with siNT or siCHC were resolved by SDS-PAGE and probed with CHC and α-actinin (loading control) antibodies. Duplicate lysates are shown. Efficiency of CHC depletion was 70±3.6% (mean±s.e.m; *n*=6). (D) mNG–VAV2 and VAV2KO cells were stimulated with 10 ng/ml EGF for the indicated times, and GTP-loaded Rac1 (Rac1-GTP) was pulled down from cell lysates with GST–PBD. Pulldowns and aliquots of lysates were resolved by SDS-PAGE and probed with anti-Rac1 and -β-actin antibodies (loading control). (E) Quantification of the amount of Rac1-GTP from three independent experiments exemplified as shown in D. The amount of Rac1-GTP (pulldowns) was corrected by the amount of β-actin in corresponding lysates. The minimum amount of corrected Rac1-GTP detected in each experiment was subtracted from corrected Rac1-GTP amounts at other time-points and variants in the same experiment. The resulting values are expressed as a percentage of the maximum Rac1-GTP amount in siNT-transfected cells in each experiment. Mean±s.e.m. (*n*=3) is presented. *P*-values for individual time-points were calculated using unpaired two-tailed Student's *t-*test.

Localization of VAV2 in EGFR-containing endosomes and the requirement of CHC, and possibly CME, for VAV2-dependent cell migration indicate that EGFR-VAV2 signaling may operate in endosomes. Small GTPase Rac1 is considered to be the major substrate of VAV2 (Bustelo, 2014). To determine whether EGFR-dependent Rac1 activity requires CME, the amount of GTP-loaded Rac1 was measured using pulldowns with a GST-fused Rac1-GTP-binding domain of PAK1 (GST–PBD) (Benard et al., 1999; Calora et al., 2003) in EGF-stimulated HSC3 cells transfected with non-targeting or CHC siRNAs. CHC knockdown did not affect initial activation of Rac1 (5-min time-point) but significantly decreased the amount of Rac1-GTP at later times (Fig. 6A–C). These data suggest that CME is required for sustained Rac1 activation when most EGFR–VAV2 complexes are in endosomes. CHC knockdown was previously shown to partially inhibit the activation of Rac1 by HGF in HeLa and MDA-MD-468 cells, although in both cases whether initial or sustained activation was inhibited is unclear because a single time after HGF stimulation was assessed (Menard et al., 2014; Palamidessi et al., 2008).

To examine whether sustained Rac1 activation requires VAV2, a Rac1-GTP pulldown assay was performed in mNG–VAV2 and VAV2KO cells stimulated with EGF for 15 and 60 min. These experiments demonstrated a significant inhibition of Rac1 activation in the absence of VAV2 (Fig. 6D,E). To confirm this observation using an alternative approach, EGF-induced Rac1 activity was shown to be strongly inhibited by RNAi-mediated knockdown of VAV2 (Fig. S5). Together, Fig. 6D,E and Fig. S5 demonstrate that VAV2 is the key GEF promoting EGF-stimulated Rac1 activity in HSC3 cells.

**Fig. 7.**
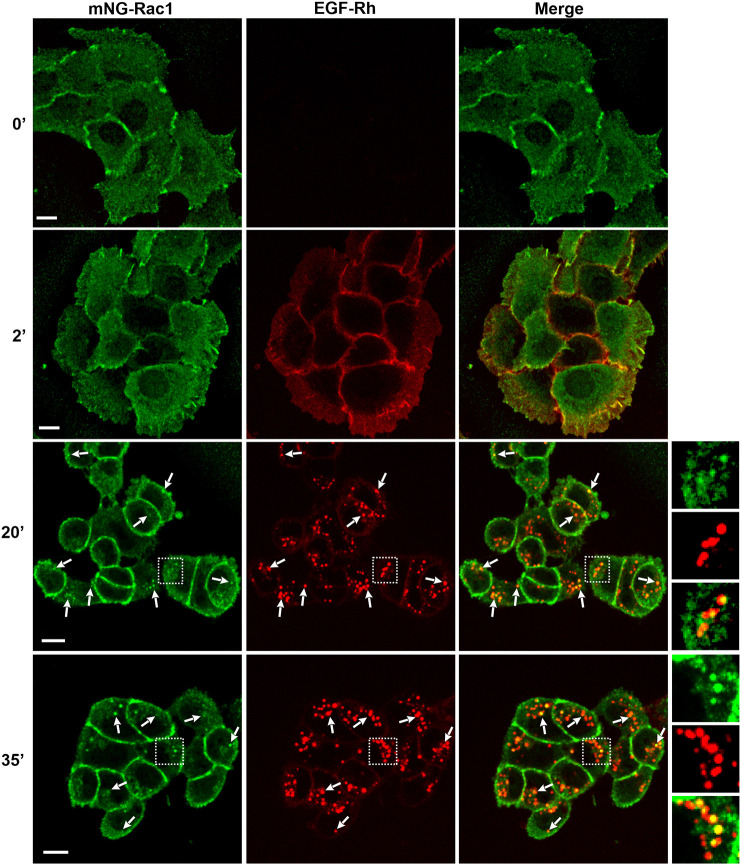
**mNG–Rac1 is localized in EGF-containing endosomes.** HSC3 mNG–Rac1 cells were serum starved and treated with 4 ng/ml EGF–Rh at 37°C for the indicated times. 3D live-cell imaging was performed through 488-nm (green, mNG-Rac1) and 561-nm (red, EGF–Rh) channels. 488-nm channel images were deconvolved using the constrained iterative algorithm of SlideBook. Maximum fluorescence intensity projections (MIPs) of two confocal sections (0 and 2 min – proximal to ventral cell membrane; and 20 and 35 min – through the middle of the z-stack) are shown. Gamma is set to 0.5 in all 488-nm channel images for better visualization of the distribution of the low-intensity mNG fluorescence. Arrows indicate examples of the vesicular puncta of EGF–Rh containing mNG-Rac1. Images on the right are high magnification images of the regions indicated by white rectangles. Images are representative of four repeats. ', minute. Scale bars: 10 μm.

The requirement of CME for sustained VAV2-dependent Rac1 activation by EGF prompted us to test whether Rac1 is present in EGFR-containing endosomes in HSC3 cells. To our knowledge, the localization dynamics of endogenous Rac1 has not been studied in living cells upon growth factor or other simulations. Available information on the tagging of endogenous Rac1 with mNG using gene-editing of HEK293 cells (https://opencell.czbiohub.org/target/607) facilitated tagging endogenous Rac1 with mNG in HSC3 cells. mNG was inserted at the N-terminus of Rac1 because such modification has been shown to not interfere with Rac1 functionality in numerous studies (for example, Kraynov et al., 2000). mNG–Rac1 was detected by western blotting with anti-Rac1 and anti-mNG antibodies in gene-edited HSC3 cells (Fig. S6). Unexpectedly, the amount of mNG–Rac1 detected by Rac1 antibody was significantly smaller than the amount of untagged Rac1 in genome-edited cells (Fig. S6). Because mNG–Rac1 was expressed in most cells in the population, the likely interpretation of the immunoblotting data is that a single allele of *Rac1* gene was edited and that either the translation of *mNG-Rac1* is not efficient or mNG–Rac1 is not efficiently recognized by Rac1 antibodies. The large amount of immunoreactivity corresponding to residual untagged Rac1 might be due to the cross-reactivity of the antibody with the highly homologous Rac2 and Rac3. Importantly, the subcellular distribution of mNG–Rac1 in HSC3 cells (Fig. 7) was essentially similar to that in HEK293 cells (https://opencell.czbiohub.org/target/607). Therefore, even though the cellular concentration of mNG–Rac1 in HSC3 cells was apparently lower than expected, these cells were further used to study Rac1 localization dynamics in EGF-stimulated cells.

In serum-starved HSC3 cells, mNG–Rac1 was diffusely distributed throughout the cell but also seen concentrated in cell contacts, edges and membrane protrusions, which is indicative of a constitutive activity of mNG–Rac1 (Fig. 7; Movie 2), although such activity appears to be significantly lower than EGF-induced activity (Fig. 6A,D). Upon EGF–Rh (4 ng/ml) stimulation, a pool of mNG–Rac1 was seen to initially concentrate in newly forming filopodia and lamellipodia, and later detected in EGF–Rh-containing endosomes (Fig. 7). The localization of mNG–Rac1 in endosomes was less obvious than the endosomal localization of mNG–VAV2, likely because the high concentration of mNG–Rac1 in the cell resulted in a strong ‘background’ of diffuse membrane and cytosolic mNG–Rac1 fluorescence. Under imaging parameter settings used in these experiments, no 488-nm channel fluorescence was detected in parental HSC3 cells indicating that mNG–Rac1 fluorescence in endosomes of genome-edited cells is specific. Clearly, only a small fraction of Rac1 is GTP-loaded (at maximum ∼1.5% based on the pulldown assay; Fig. 6A). We have not been able to detect endogenous Rac1 activity in HSC3 cells using the fluorescence-based sensors characterized in Pimenta et al. (2023). Nevertheless, mNG–Rac1 could be found in EGF–Rh-containing endosomes in the majority of cells in the population. Localization of mNG–Rac1 in EGF–Rh-containing endosomes was much more pronounced in cells treated with 40 ng/ml EGF–Rh (Fig. S7), presumably, because of an increased amount of EGFR:VAV2 complexes in these endosomes (Fig. S2).

## DISCUSSION

Our study provides four lines of evidence supporting the hypothesis of EGFR-VAV2-Rac1-mediated cell motility signaling from endosomes in HSC3 cells. First, endogenous VAV2 is associated with endosomal EGFR for an extended time after EGF stimulation. Second, EGFR- and VAV2-dependent cell migration is clathrin dependent. Third, sustained VAV2-dependent activation of Rac1 also requires clathrin. Finally, endogenous Rac1 is present in EGFR-containing endosomes. In similarity with our studies in living cells, the endosomal localization of endogenous Rac1 has been demonstrated by immunofluorescence staining of HGF-stimulated HeLa (Palamidessi et al., 2008) and MDA-MD-468 cells (Menard et al., 2014). However, the proposed roles and mechanisms of c-MET endosomal signaling to Rac1 are different in these two studies. The former study implicated TIAM-1, another Rac1 GEF and a Rab5 effector, in endocytosis- and Rab5-dependent signaling from c-MET to Rac1 and cell migration (Palamidessi et al., 2008), whereas Menard et al. demonstrate that VAV2 but not TIAM-1 is involved in Rac1 activation by HGF (Menard et al., 2014). Certainly, the contribution of different GEFs in Rac1 activation is cell type dependent (Itoh et al., 2008; Marignani and Carpenter, 2001). In HSC3 cells, our data indicate that VAV2 is essential for EGF-induced Rac1 activation (Fig. 6D,E; Fig. S5) and has a major role in EGF-guided chemotactic cell migration (Fig. 4B,C).

Thus, in contrast to the EGFR-Grb2-SOS-Ras signaling axis, all components of the EGFR-VAV2-Rac1 axis are present in endosomes, and the sustained activation of a GTPase as well as the signaling outcome (cell migration) are clathrin dependent. Certainly, this signaling pathway operates at the plasma membrane in HSC3 cells as evident from the initial translocation of VAV2 and Rac1 to filopodia and lamellipodia (Figs 2 and 7), and from clathrin-independent Rac1 activation early after cell stimulation with EGF (Fig. 6A,B). Whether the function of EGFR-VAV2-Rac1 signaling in endosomes is different than in the plasma membrane is unclear. In the case of c-MET-mediated activation of Rac1, it has been suggested that active Rac1 translocates from endosomes to the plasma membrane to promote lamellipodia formation and cell migration (Palamidessi et al., 2008; Menard et al., 2014). Such translocation was not observed in mNG–Rac1 HSC3 cells in our live-cell imaging studies, although the sensitivity of the detection of Rac1 traffic was limited due to a strong background of the diffuse mNG–Rac1 fluorescence. Menard et al. proposed a specific role of Rac1 localization in perinuclear endosomes in its downstream signaling (Menard et al., 2014), although it remains unclear what is ‘specific’ about these endosomes as a signaling platform. The contribution of different endosomes and endosomal signaling in the RTK-GEF-Rac1 pathway might depend on the kinetics of endocytic trafficking of an RTK. In cells like HSC3, expressing high levels of EGFR, sorting to late endosomes and lysosomes is relatively slow, resulting in a prolonged residence time of active EGFR and mNG–VAV2 in early endosomes (Fig. 3). At the same time, in HSC3 cells exposed to physiological EGF concentrations, the residence time of active EGFR at the cell surface is short due to rapid CME. Therefore, we propose that the activity of the EGFR-VAV2-Rac1 pathway in endosomes serves to prolong and sustain signaling rather than producing a qualitatively different outcome than that from signaling by plasma membrane EGFR.

## MATERIALS AND METHODS

### Reagents

Recombinant human EGF was purchased from BD Biosciences. EGF–Rh was purchased from Molecular Probes. HGF was provided by Dr George K. Michalopoulos (University of Pittsburgh, USA). Antibodies against GAPDH (#2118), EGFR (#4267), Akt (#9272), EGFR pTyr992 (#2235), EGFR pTyr1173 (#4407) and α-actinin (#6487) were from Cell Signaling Technology. Antibodies against VAV2 (sc-271442) and β-actin (sc-8432) were from Santa Cruz Biotechnology. Anti-pY20-HRP antibody (610012) was from BD Transduction Laboratories. Anti-Rac1 antibody was from Cytoskeleton Inc (ARC03). Antibody against CHC (ab21679) was purchased from Abcam. Monoclonal antibody to EGFR (#05-104) was from Millipore. Secondary anti-mouse-IgG (catalog nos. 926-32220, 926-32210 and 926-32350) and anti-rabbit-IgG (catalog nos. 926-32211 and 926-68071) IRDye-labeled antibodies were from LI-COR. Primary and secondary antibodies were used at 1:1000 and 1:20,000 dilution, respectively. All oligonucleotides listed in Table S1 were purchased from Integrated DNA Technologies (IDT). Lipofectamine transfection reagents were from Thermo Fisher Scientific, whereas JetOptimus transfection reagent was purchased from Polyplus. PCR and cloning reagents were purchased from New England Biolabs (NEB). Nocodazole was purchased from Sigma Chemicals. Phalloidin-TxR was from Invitrogen (#T7471).

### Generation of mNG–VAV2 and VAV2-knockout HSC3 cells

VAV2 was tagged at its N-terminal with the full-length mNeonGreen (mNG) fluorescent protein using CRISPR/Cas9 technology. Two gRNA targets were selected using various gRNA design algorithms (Table S1). A gRNA was obtained in the form of ssDNA from IDT (ssDNA VAV2-Grna_T1) and was cloned in PX459 (Addgene #62988) using HiFi DNA assembly mix and confirmed using Sanger sequencing.

To generate double stranded donor DNA for knock-in of VAV2, the mNG full-length sequence was flanked with 5′ and 3′ homology arms (HA). First, a ∼1200 bp PCR amplicon was generated using Vav5HA-F2 and Vav3HA-R2 primers from HSC3 genomic DNA in the presence of 7% DMSO. The NEBuilder algorithm was employed to generate primers that add overlapping sequences to the donor DNA fragments. Using an ∼1200 bp amplicon as a template 5′HA (854 bp) was generated using 5HA_fwd and 5HA_rev primers. 3′HA (453 bp) was generated using 3HA_fwd and 3HA_rev primers. mNG PCR amplicon (755 bp) was generated using mNG_fwd and mNG_rev primers and mNG donor DNA cloned previously (Surve et al., 2021) as template. 5′HA, mNG and 3′HA were cloned in a linear Halo EasyFusion vector using HiFi DNA assembly mix. The final double stranded mNG–VAV2 donor DNA clone was confirmed using Sanger Sequencing.

HSC3 cells (see below) were treated with nocodazole (100 ng/ml) before transfection with PX459-VAV2 gRNA T1 and mNG–VAV2 donor DNA using JetOptimus Reagent at a ratio of 1:3, respectively. The transfection mixture was removed after 6 h and the cells were allowed to recover in fresh DMEM with 5% FBS. The cells were expanded, and mNG-positive cells were sorted as pooled cells. mNG–VAV2 cells could not be grown as single-cell colonies and therefore were maintained and stored frozen as pooled clone populations. Pooled clones of mNG-positive cells were stimulated with EGF–Rh and analyzed using confocal microscopy. To this end, cell pools with the accumulation of mNG fluorescence in nuclei along with the diffuse cytoplasmic distribution, a pattern typical of the distribution of unfused fluorescent proteins, were discarded, whereas cell pools in which mNG displayed cytoplasmic and endosomal localization were selected followed by their validation by western blotting.

To generate VAV2KO cells, mNG–VAV2 cells were transfected with gRNA targeting exon 5 of the *VAV2* gene. exon 5 VAV2 gRNA was obtained in the form of ssDNA and cloned in PX459 using HiFi DNA assembly mixture. The clone was confirmed using Sanger sequencing. mNG–VAV2 cells were transfected with 1 µg PX459-Exon 5 gRNA and treated with 2 µg/ml puromycin for 2 days to eliminate non-transfected cells. The cells negative for mNG fluorescence were sorted out and the absence of VAV2 in VAV2KO cells was confirmed by western blotting (Fig. 3A).

### Generation of HSC3 cells expressing endogenous mNG–Rac1

Rac1 was tagged with full-length mNG at its N-terminus using CRISPR/Cas9. Two gRNAs for Rac1 knock-in were selected using Benchling and ordered in the form of a ssDNA, rac-gRNA_1 and rac-gRNA_2 (Table S1). The gRNAs were cloned in PX459 (Addgene #62988) using HiFi DNA assembly mix (NEB) and confirmed using Sanger sequencing.

To generate double-stranded donor DNA for knock-in of Rac1, the mNG full-length sequence was flanked with 5′ and 3′ HAs. First, a ∼1000 bp PCR amplicon was generated using RacF1 and RacR1 primers from HSC3 genomic DNA in the presence of 5% DMSO. The NEBuilder algorithm was employed to generate primers that add overlapping sequences to the donor DNA fragments. Using ∼1000 bp amplicon as a template 5′HA (487 bp) was generated using Rac5HA_fwd and Rac5HA_rev primers. Rac3′HA (489 bp) was generated using Rac3HA_fwd and Rac3HA_rev primers. mNG PCR amplicon (755 bp) was generated using mNG_fwd and mNG_rev primers and mNG donor DNAs cloned previously (Surve et al., 2021) as template. 5′HA, mNG and 3′HA were cloned in a linear Halo EasyFusion vector using HiFi DNA assembly mix. The final double-stranded mNG–Rac1 donor DNA clone was confirmed using Sanger Sequencing.

HSC3 cells were treated with nocodazole (100 ng/ml) before transfection with PX459-Rac1 gRNAs and mNG–Rac1 donor DNA using JetOptimus Reagent at a ratio of 1:3, respectively. Transfected cells were expanded to sort mNG-positive cells. The sorted cells were grown as pooled cells. Pooled mNG-positive cells were validated using confocal microscopy for the plasma membrane localization of mNG–Rac1 and by western blotting (Fig. S6). The pooled HSC3 mNG–Rac1 cells were also validated by sequencing to verify there was no change in the reading frame of Rac1 at the site of insertion of mNG. Pool #1 (Fig. S6) was used in experiments.

### Cell culture

HSC3 cells (provided by the University of Colorado Cancer Center) and its gene-edited variants were maintained in Dulbecco's minimum essential medium (DMEM) supplemented with 5% fetal bovine serum (FBS) (Invitrogen). The identity of HSC3 cells was confirmed by genotyping, and cells were tested for mycoplasma monthly.

### RNA interference

Cells were grown in six-well plates at 60% confluency and transfected with either non targeting, CHC siRNA (duplex 2; Table S1; Dharmacon/Horizon Discovery) (Huang et al., 2004) (50 nM final concentration) or Flextube VAV2 siRNA (Qiagen; #S102662947) (50 nM final concentration) using Dharmafect Reagent 1(Horizon Discovery). The cells were used after 3 days in cell migration and spreading assays. Cells for the GST–PBD pulldown assays were grown in 100 mm plates and transfected with siRNAs on the next day and 2 days after. Cells were re-plated according to experimental design, serum-starved overnight and assayed 2 days after the second transfection.

### Immunoprecipitation and western blotting

Parental HSC3 and mNG–VAV2 cells were plated in 100 mm plates. The cells were serum-starved overnight and stimulated with 4 ng/ml EGF for 5 and 20 min. The cells were washed with cold PBS and lysed in 300 µl Triton X-100 (1%), glycerol (10%) and 20 mM HEPES (TGH) buffer supplemented with 150 mM NaCl, 2 mM EDTA, 2 mM EGTA, 1 mM phenylmethylsulfonyl fluoride, 1 mM sodium orthovandate, 10 mM N-ethylmaleimide, 10 µg/ml aprotinin, 10 µg/ml leupeptin and protease inhibitor cocktail (Sigma-Aldrich). The lysates were cleared by centrifugation at 14,000 rpm (11,000 ***g***) at 4°C. VAV2 and mNG–VAV2 were immunoprecipitated with 1.5 µg of VAV2 antibody by incubating overnight at 4°C followed by pulldown with Sepharose G beads (Thermo Fisher Scientific #10-1243) for 1.5 h at 4°C and denaturing at 95°C in sample buffer. Cell lysates and immunoprecipitates were resolved by SDS-PAGE and transferred to nitrocellulose following by western blotting as described previously (Surve et al., 2021). Detection was performed by LI-COR or chemiluminescence with SuperSignal West Dura Extended Duration Substrate (Thermo Fisher Scientific #34075) for pY20-HRP antibody.

### Live-cell fluorescence microscopy imaging

The cells were plated in 35 mm Mat-Tek dishes. Overnight serum-starved cells were stimulated with 4 ng/ml EGF–Rh. Live-cell 3D imaging was performed using a spinning disk confocal Marianas system based on Zeiss Axio Observer Z1 inverted fluorescence microscope and CSU-W1 spinning disk, equipped with 405-, 445-, 488-, 515-,561- and 640-nm lasers, a 63×1.4 NA oil immersion objective, EM-CCD cameras (Photometrics Evolve was recently replaced by Andor iXon) and a piezo-controlled *z*-step motor, all controlled by SlideBook software (Intelligent Imaging Innovation). The imaging was performed at 37°C and 5% CO_2_ in an environmental chamber using *z*-stacks of 15–20 *x-y* confocal images with 0.3 µm steps. The images were analyzed using Slidebook software.

For imaging of endosomal markers, starved mNG–VAV2 cells were pre-incubated for 5 min with LysoTrackerBlue (Invitrogen; dilution 1:5000 in medium) and 5 µg/ml Trf–A647 (Invitrogen); and then stimulated with EGF–Rh (4 ng/ml) in the presence of Trf–A647.

To quantify the amount of mNG–VAV2 colocalized with EGF–Rh in endosomes, background-subtracted 3D images of cells stimulated with EGF–Rh for more than 10 min (at the time when the intensity of EGF–Rh fluorescence in endosomes is higher than at the plasma membrane) were used to generate a segment mask to select vesicular compartments (EGF–Rh-containing endosomes) detected through the 561-nm channel (Mask-561). A few objects overlapping with the plasma membrane were removed in each *x-y* image of *z*-stack manually. Another segment mask was generated with the minimum threshold to include all voxels detected through the 488-nm channel (total mNG–VAV2; Mask-488). For both masks, identical threshold parameters were used for experimental variables within each individual time-course experiment. The sum fluorescence intensity of the 488-nm channel in the 561 nm mask was divided by the sum fluorescence intensity of Mask-488 in each field of view (FOV) with multiple cells to calculate the fraction of total cellular mNG–VAV2 colocalized with EGF–Rh. The ratio of the sums of fluorescence intensities of 488 nm and 561 nm channels was also calculated per FOV in mNG–VAV2 and parental HSC3 cells treated with EGF–Rh using Mask-561 (Fig. S1).

To quantify the fraction of mNG–VAV2 colocalized with EGF–Rh in early and sorting endosomes versus late endosomes and lysosomes, Mask-561 and Mask-488 were generated as described above. A colocalization mask (MaskEGF+VAV2) was then generated by selecting voxels overlapping in Masks 561 and 488. The ‘Trf’ and ‘LT’ masks were generated to select voxels positive for Trf–A647 or LysotrackerBlue, respectively. An Trf-LT mask was generated that contained voxels in which Trf–A647 or LysotrackerBlue were colocalized (sorting endosomes). To generate a mask corresponding to late endosomes and lysosomes (‘LEL’ mask; defined as vesicles not containing Trf–A647), the Trf+LT mask was subtracted from the LT mask. The ratio of the sum of mNG–VAV2 fluorescence intensity in the Trf mask (early plus sorting endosomes) and the LEL mask to the sum of mNG–VAV2 fluorescence intensity in MaskEGF+VAV2 (total endolysosomal mNG–VAV2) corresponded to the fraction of vesicular mNG-VAV2 in early and sorting endosomes and late endosome and lysosomes, respectively.

### Cell spreading assay

Serum-starved parental HSC3 and VAV2KO cells were plated on coverslips coated with fibronectin (Sigma-Aldrich #1141–20 mg/ml) in serum-free medium containing 0.1% BSA and 4 ng/ml EGF. The cells were allowed to adhere to the coverslip and spread for 1 h and fixed with freshly prepared 4% paraformaldehyde (PFA) in PBS. Fixed cells were permeabilized with 0.1% Triton X-100 for 5 min, washed with PBS, and stained with Phalloidin–TxR (1:2000 diluted in PBS) for 15 min at room temperature, mounted in ProlongGold (Invitrogen) and 3D-imaged using a spinning disk microscope. For quantification of cell spreading efficiency, a 561 mask was generated from the maximum fluorescence intensity projections of 3D images, and the footprint (total area of positive pixels per cell) of individual cells on the substrate was measured.

### Boyden Chamber migration assay

Boyden Chamber migration assays were carried out in sterile tissue culture plate inserts with polycarbonate membranes (VWR, Cat #10769-234). The membrane side facing the bottom of the 24-well plate was coated with fibronectin before plating 10^5^ serum-starved cells in the top chamber of the inserts. The bottom chamber was filled with DMEM containing 0.1% BSA and 4 ng/ml EGF. Migration was allowed to take place for 4 h at 37°C. Unattached cells from the top chamber were removed by suction, and cells on the top and bottom surfaces of the chamber were fixed in PFA for 15 min. The cells were stained with 0.1% Crystal Violet (Invitrogen) for 20 min and washed several times with water to remove excess stain. Cells from the top surface of the chamber were removed using a cotton swab. Migrated cells were then counted using 10× objective from 10 randomly chosen FOVs. The researcher was aware of the experimental conditions while counting.

### Rac1-GTP GST pulldown assay

A GST-fused Rac1-GTP binding fragment comprising amino acids 70–117 of PAK1 (GST–PBD) in pGEX-2T was purchased from Addgene (#12217). Bacterially expressed GST–PBD was pulled down from precleared lysates using glutathione–Sepharose 4B beads (Cytiva, Sweden). The beads were washed in Ca^2+^- and Mg^2+^-free phosphate buffer saline and immediately used in experiments. siNT- or siCHC-transfected HSC3 cells were serum starved overnight and stimulated with EGF (10 ng/ml). The cells were washed with ice-cold Dulbecco's PBS (DPS; Gibco) and solubilized in lysis buffer [1% Triton X-100, 150 mM NaCl, 20 mM MgCl_2_, 50 mM Tris-HCl, pH 8, phosphatase and protease inhibitors (Sigma-Aldrich)]. GST–PBD beads were incubated with 0.8–1.0 mg of precleared cell lysates for 1 h at 4°C. After extensive washes of beads with lysis buffer, proteins were eluted from beads in 2× sample buffer and heated for 5 min at 95°C. GST pulldowns and aliquots of cell lysates were resolved by SDS-PAGE using 4–20% gradient (for better separation of specific Rac1 bands) or 10% acrylamide gels, respectively, transferred to nitrocellulose membranes, and the membranes were probed by western blotting with Rac1 and β-actin antibodies. Secondary antibodies conjugated to infrared fluorescent dyes (LI-COR Biosciences) were used, and blots were imaged and analyzed using an Odyssey Infrared Imaging system (LI-COR Biosciences).

### Statistical analysis

All statistical analyses were performed using GraphPad Prism software. For comparisons of each two groups, an unpaired two-tailed Student's *t-*test was used, and for comparisons of more than two groups, one-way analysis of variance (ANOVA) followed by Tukey's multiple comparison test was used. Biological repetition numbers are indicated on the graphs or in figure legends. Differences were considered significant when the *P*<0.05.

## Supplementary Material



10.1242/joces.263541_sup1 Supplementary information
