# Supplementary material for: Cell migration signaling through the EGFR-VAV2-Rac1 pathway is sustained in endosomes
Source: J Cell Sci. 2025 Jan 24;138(2):jcs263541. doi: 10.1242/jcs.263541 (PMC11828472; doi:10.1242/jcs.263541)
Supplement: Supplementary information [file joces-138-263541-s1.pdf]

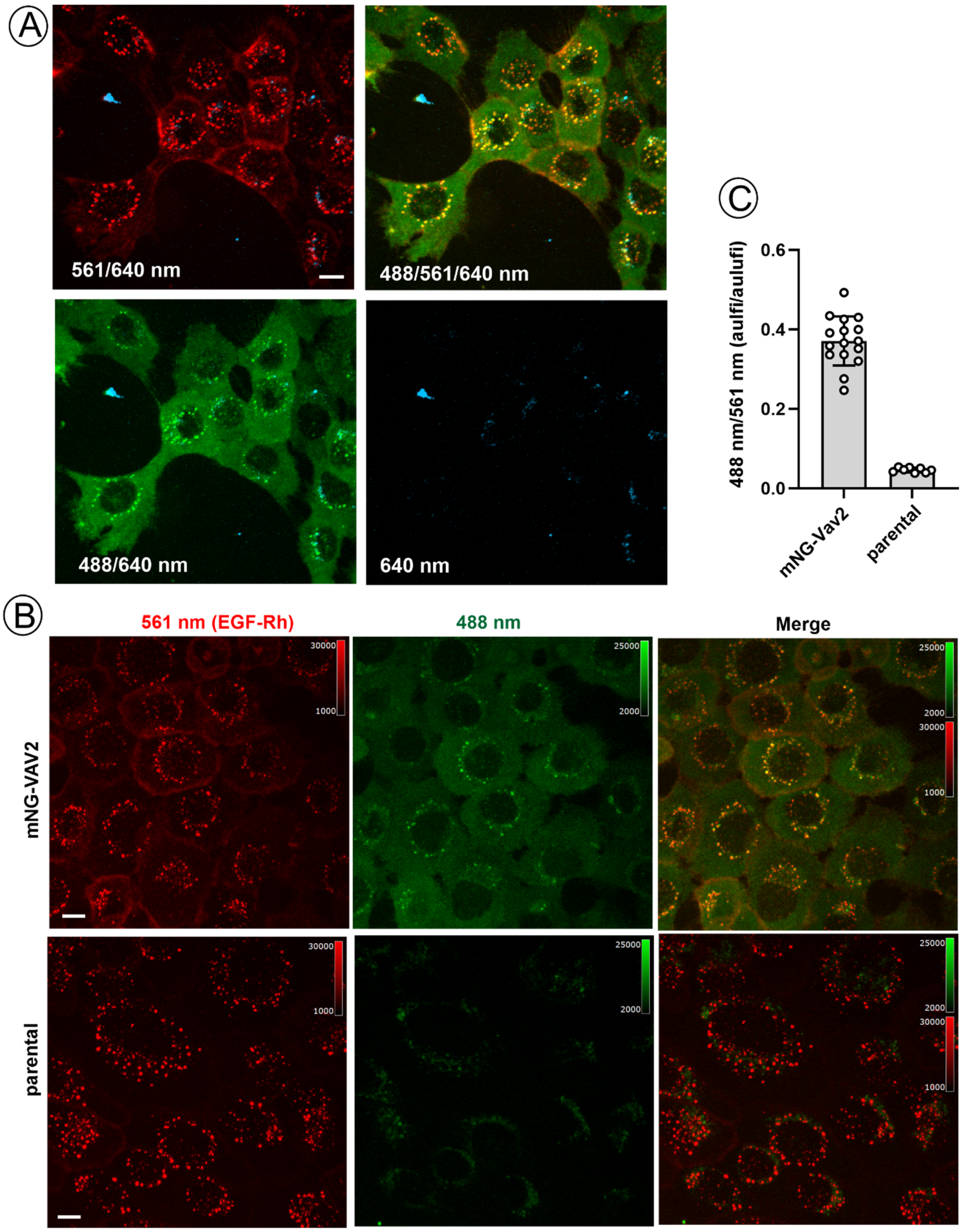
A
561/640 nm
488/561/640 nm
488/640 nm
640 nm
C
488 nm/561 nm (aulfi/aulufi)
0.6
0.4
0.2
0.0
mNG-Vav2
parental
B
561 nm (EGF-Rh)
488 nm
Merge
mNG-VAV2
parental
30000
1000
25000
2000

Fig. S1. Fluorescence detected through the 488 nm channel in EGF-Rh-containing endosomes in mNG-VAV2 cells reports mNG-VAV2 localization.

(A) Comparison of the localization of EGF-Rh:mNG-VAV2 fluorescence and non-specific fluorescence detected through the 640-nm channel in the 20-min timepoint image presented in Fig. 2. Fluorescence intensity scales of 488 nm and 561 nm channels are the same as in Fig. 2. Because of the low intensity signal (low signal-to-noise over background) detected through the 640 nm channel, the intensity scale of the 640-nm fluorescence was set 10-times smaller than that of the 488-nm channel fluorescence. Scale bar, 10 μm.

(B) mNG-VAV2 and parental HSC3 cells were serum starved and incubated with 4 ng/ml EGF-Rh at 37°C for 25 min. 3D live-cell imaging was performed through 488-nm (*green*) and 561-nm (*red*) channels using identical parameter settings. Fluorescence intensity scales are shown. Individual confocal sections are shown. Scale bars, 10 μm.

(C) Quantification of the ratio of the fluorescence intensities through 488 nm and 561 nm channels in EGF-Rh-containing endosomes in mNG-VAV2 and parental HSC3 cells incubated with EGF-Rh for 10-30 min as in ***A***. Fluorescence intensities were measured as arbitrary units of linear fluorescence intensity (a.u.l.f.i). Each data point represents a single FOV. $P<0.0001$ was calculated using T-test.

Journal of Cell Science • Supplementary information

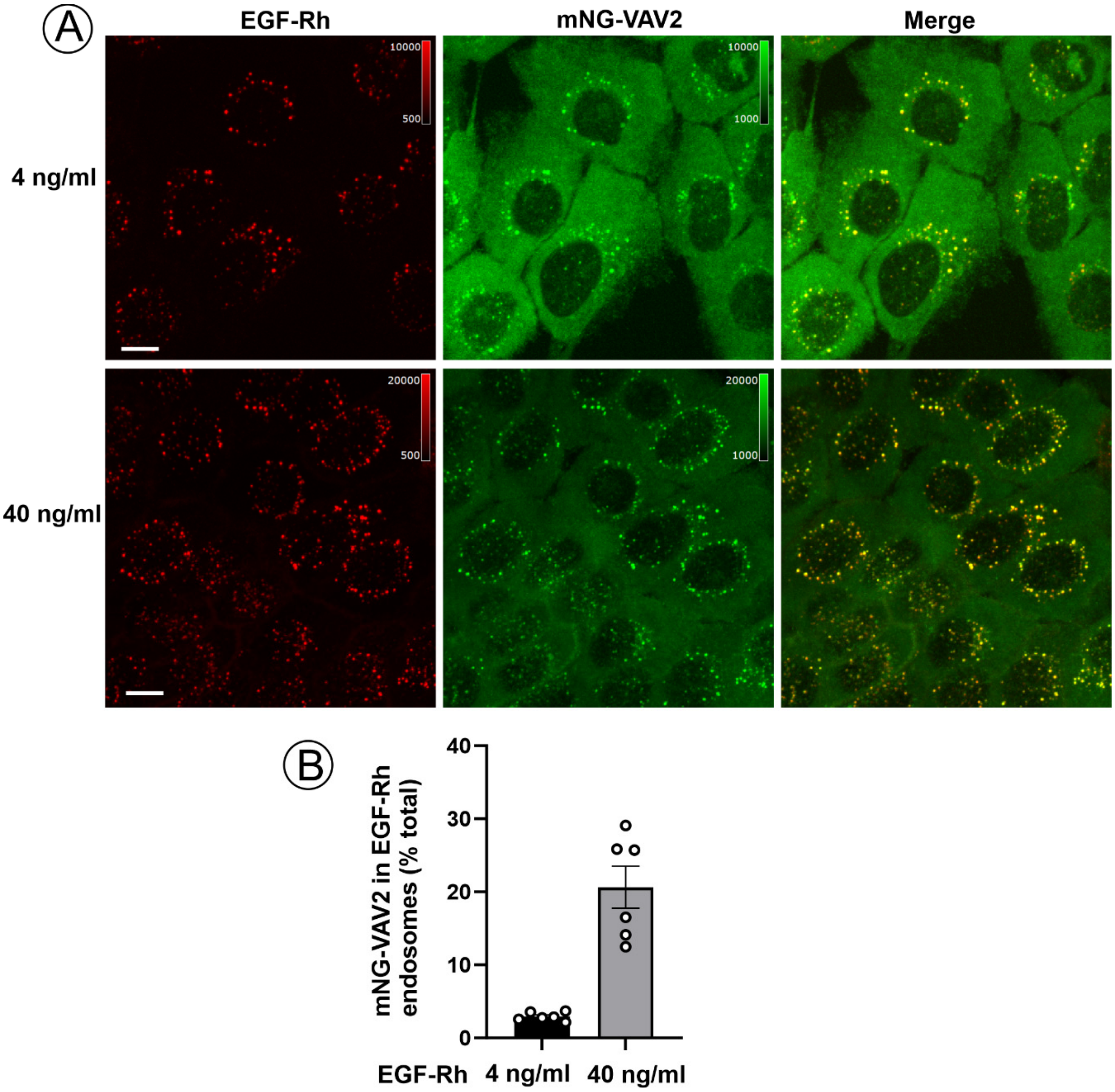

Fig. S2. Increased colocalization of endogenous mNG-VAV2with EGF-Rh in endosomes in cells stimulated with high (40 ng/ml) EGF-Rh concentration.

(A) mNG-VAV2 cells were serum starved and incubated with 4 or 40 ng/ml EGF-Rh at 37°C for 15 min. 3D live-cell imaging was performed through 488-nm (*green*; mNG-VAV2) and 561-nm (*red*; EGF-Rh) channels. Maximum intensity projections (MIP) are shown. Note different intensity scales in 4 ng/ml versus 40 ng/ml EGF-Rh images. Scale bar, 10 μm.

(B) Quantification of the fraction of mNG-VAV2 in endosomes containing EGF-Rh. Mean values from 6 FOVs are presented. P<0.0001 per T-test.

Journal of Cell Science • Supplementary information

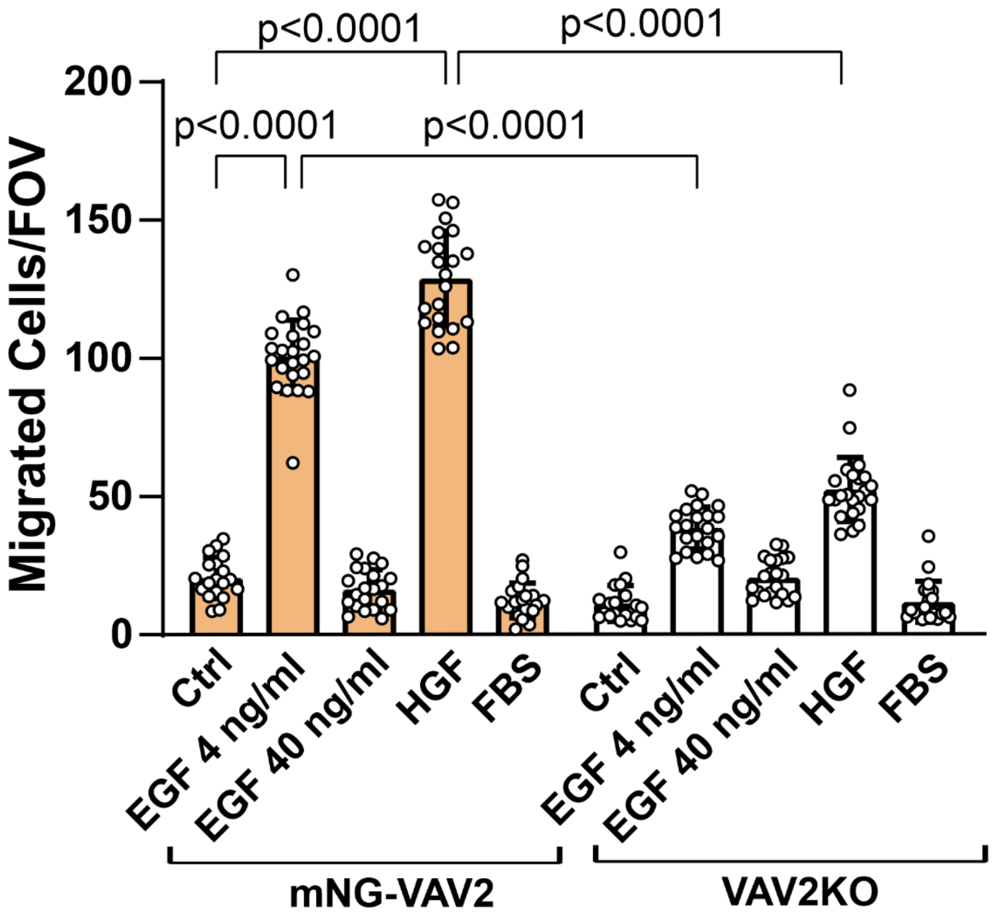

Fig. S3. Chemotactic migration of mNG-VAV2 and VAV2KO cells towards EGF, HGF or FBS.

mNG-VAV2 and VAV2KO cells were plated in the upper chamber of pre-treated Transwell inserts (10^5 cells/insert). Media with or without 4 ng/ml or 40 ng/ml EGF, 20 ng/ml HGF or 10%FBS was added to the bottom compartment of wells. Cells were incubated for 4 hrs at 37°C to allow cell migration to the bottom surface of Transwell inserts, fixed and stained with DAPI. Cells migrated to the bottom surface of Transwell inserts were imaged. Bar graphs represent mean numbers of cells per FOV (±S.E.M; 6 FOVs per insert; n = 2 inserts). The data are from two independent experiments. P values were calculated using one-way ANOVA. Differences between controls (ctrl), 40 ng/ml EGF and FBS are not significant (p>0.05).

Journal of Cell Science • Supplementary information

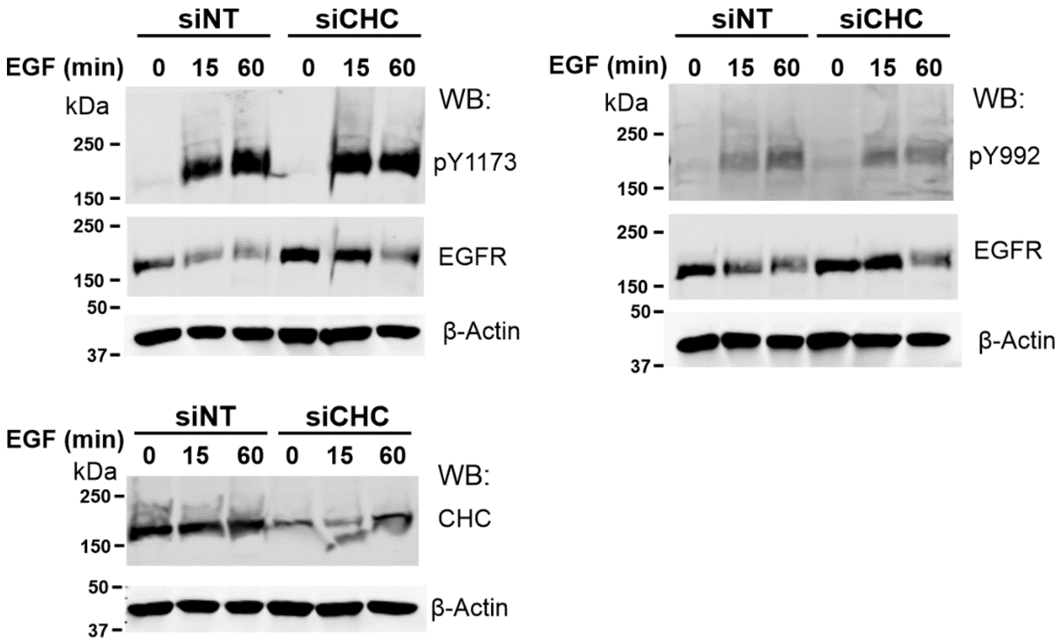

Fig. S4. Phosphorylation of EGFR at Tyr992 and Tyr1173 is not notably affected by CHC knockdown.

mNG-VAV2 and VAV2KO cells were serum starved and stimulated with 4 ng/ml EGF for 0, 15 and 60 min at 37°C. The cells were lysed, and three equal aliquots of lysates were electrophoresed and probed by Western blotting with antibodies to pY992, pY1173, EGFR (05-104), CHC (knockdown control) and β-actin as loading control.

Journal of Cell Science • Supplementary information

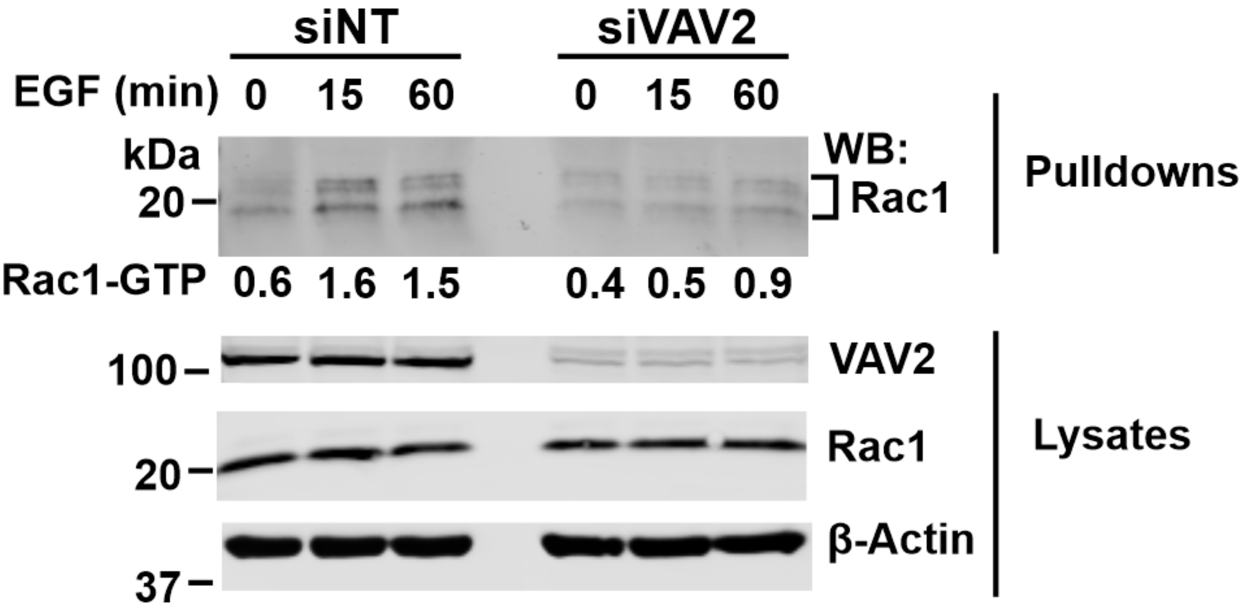

Fig. S5. VAV2 is required for sustained Rac1 activation by EGFR.

Parental HSC3 cells were transfected with non-targeting (siNT) and VAV2 targeting siRNA (siCHC). The cells were stimulated with 10 ng/ml EGF for indicated times, and GTP-loaded Rac1 (Rac-GTP) was pulled down from cell lysates with GST-PBD. Pulldowns and aliquots of lysates were resolved by SDS-PAGE and probed with Rac1 and β-actin antibodies (loading control). The amounts of Rac1-GTP in pulldowns corrected by the amounts of β-actin in lysates were quantitated and presented in arbitrary units.

Journal of Cell Science • Supplementary information

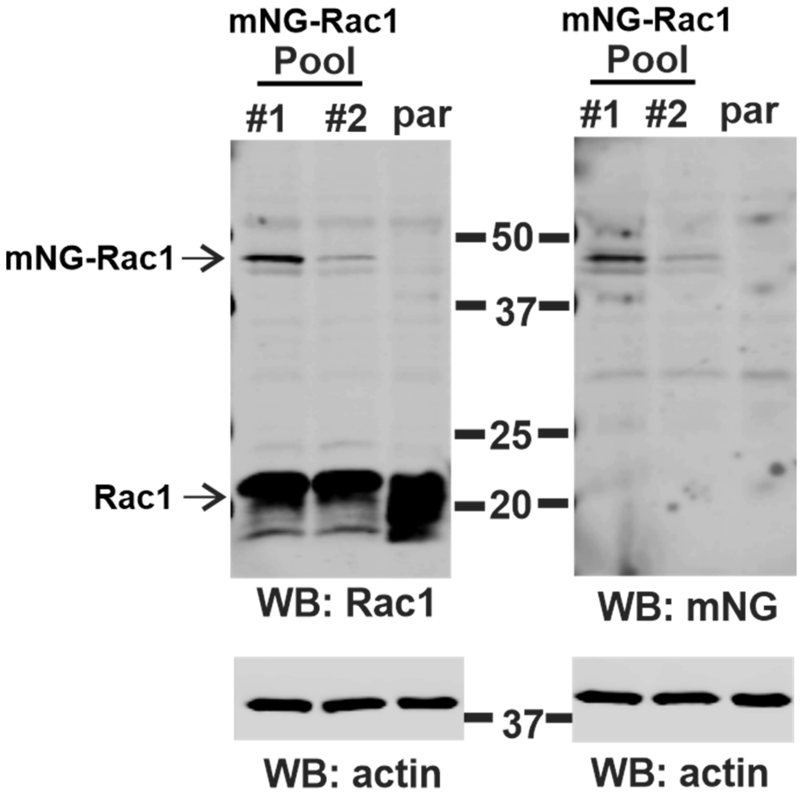

Fig. S6. Western blotting analysis of mNG-Rac1/HSC3 genome-edited cells.

Lysates of parental (par) HSC3 and two pools of genome edited HSC3/mNG-Rac1 cells were probed by Western blotting with Rac1, mNG and β-actin (loading control). Pool #1 was used in experiments. The correct insertion of *mNG* with the linker GGGSAAA at the amino-terminus of *Rac1* was confirmed by PCR.

Journal of Cell Science • Supplementary information

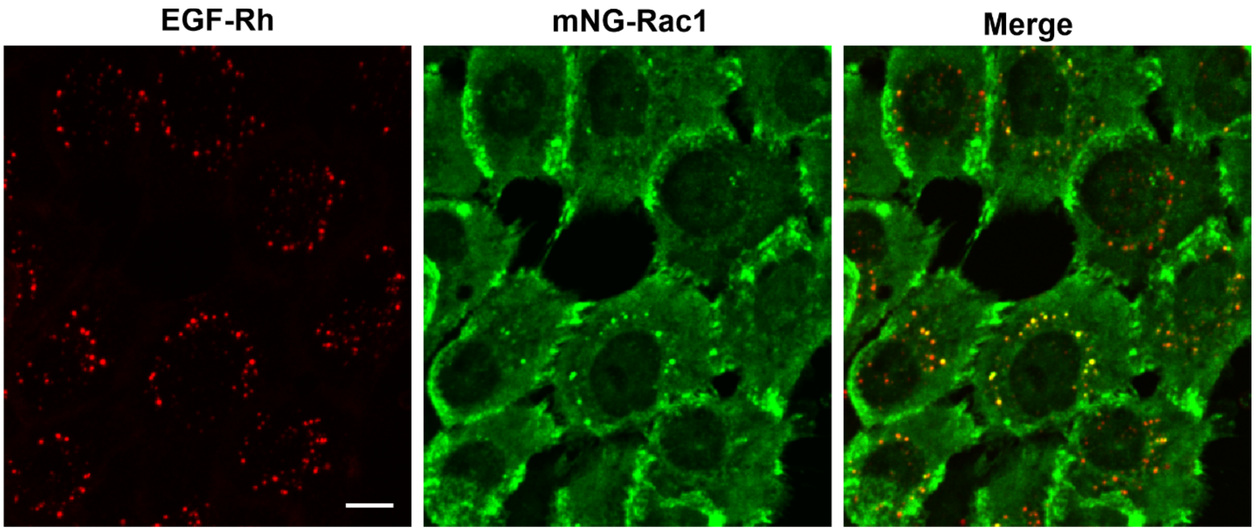

Fig. S7. Localization of endogenous mNG-Rac1 in cells stimulated with high EGF-Rh concentration (40 ng/ml).

HSC3/mNG-VAV2 cells were serum starved and incubated with 40 ng/ml EGF-Rh at 37°C for 15 min. 3D live-cell imaging was performed through 488-nm (*green*; mNG-Rac1) and 561-nm (*red*; EGF-Rh) channels. 488-nm channel images were deconvolved using constrained iterative algorithm of SlideBook. MIPs of 10 consecutive confocal planes are shown. Gamma is set to 0.6 in 488-nm channel images for better visualization of the low-intensity mNG fluorescence. Scale bar, 10 μm.

Table S1. Oligonucleotides, siRNAs, gRNAs and donor ssDNAs for CRISPR/Cas9

Oligonucleotide	Sequence 5' – 3'
ssDNA Vav2-Grna_T1	ATCTTGTGGAAAGGACGAAACACCGGGGTCGGGCCCGCGGGCGCCAGTTTTAGAGCTAGAAATAGCAAGTT
Vav5HA-F2	CCTCCACGGAGGACCAAA
Vav3HA-R2	CCCAGGCTGAGGGGACTT
5HA_fwd	TATCGATAAGCTTGATATCGCCTCCACGGAGGACCAAAGGCACCCGG
5HA_rev	TGCTCACCATGGCGCCCGCGGGCCCGAC
3HA_fwd	AGCAGCAGCAATGGAGCAGTGGCGGCAG
3HA_rev	CCACCGCGGTGGCGGCCGCTCGCGACCCAGGCTGAGGG
mNG_fwd	CGCGGGCGCCATGGTGAGCAAGGGCGAG
mNG_rev	ACTGCTCCATTGCTGCTGCTGATCCTCC
ssDNA Exon5_Vav2	ATCTTGTGGAAAGGACGAAACACCGGGTGGAGGGGACGACATCTACGGTTTTAGAGCTAGAAATAGCAAGTT
rac-gRNA_1	ATCTTGTGGAAAGGACGAAACACCGGGACACTTGATGGCCTGCATCAGTTTTAGAGCTAGAAATAGCAAGTT
rac-gRNA_2	ATCTTGTGGAAAGGACGAAACACCGGGATGCAGGCCATCAAGTGTGGTTTTAGAGCTAGAAATAGCAAGTT
RacF1	CGAGCTGACAGGTTCCTCTT
RacR1	GTACAATCAAGCATGCATCAAA
Rac5HA_fwd	TATCGATAAGCTTGATATCGCGAGCTGACAGGTTCCTCTTAAAGCCCTG
Rac5HA_rev	TGCTCACCATCAGGGCCGCTCGCTGGGC
Rac3HA_fwd	AGCAGCAGCAATGCAGGCCATCAAGTGTG
Rac3HA_rev	CCACCGCGGTGGCGGCCGCTGTACAATCAAGCATGCATCAAATATG
mNG_fwd	AGCGGCCCTGATGGTGAGCAAGGGCGAG
mNG_rev	TGGCCTGCATTGCTGCTGCTGATCCTCC
siRNA CHC	GCAATGAGCTGTTTGAAGA

Journal of Cell Science • Supplementary information

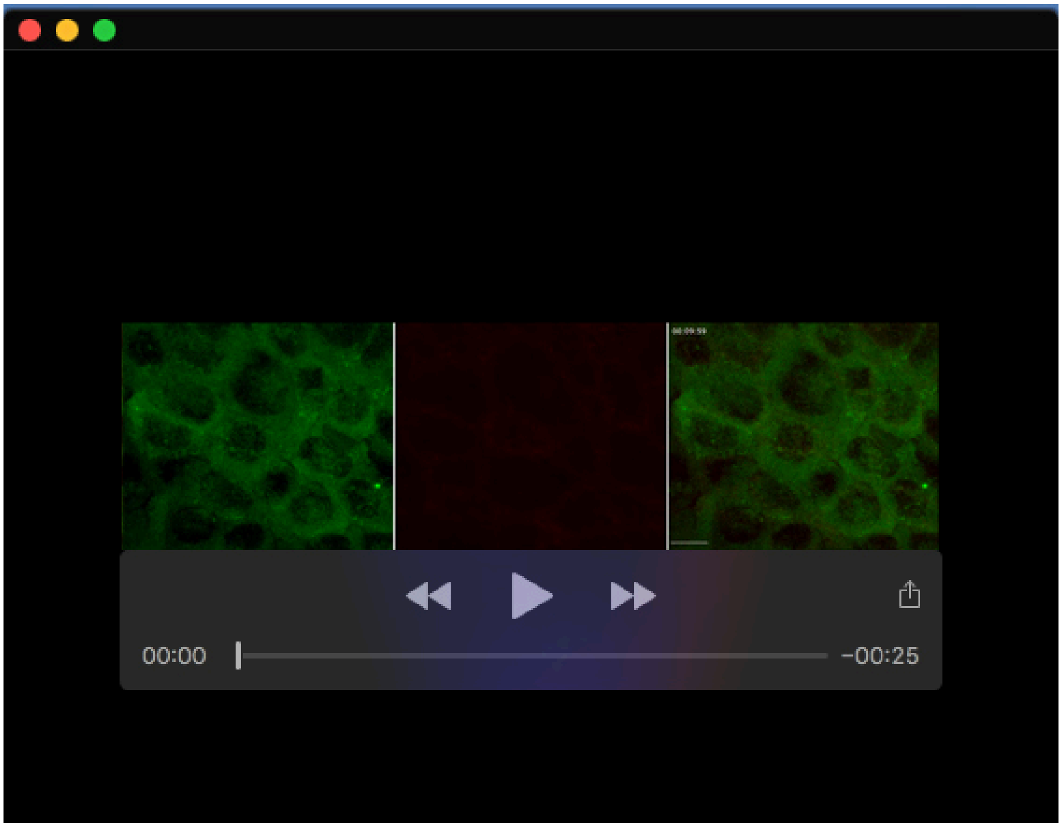

Movie 1. mNG-VAV2 cells were serum starved, placed onto microscope stage into environmental chamber, and 3D live-cell images were acquired through 488-nm (*green*) and 561-nm (*red*) channels from living cells at 37°C for 45 min. EGF-Rh (final concentration 4 ng/ml) was perfused 3 min after the start of time-lapse imaging. Imaging parameters for acquiring EGF-Rh images were used to avoid saturating signals at later time points, which resulted in the lack of visible EGF-Rh fluorescence at early time points. MIP images of all 15 planes are presented. Scale bar, 10 μm.

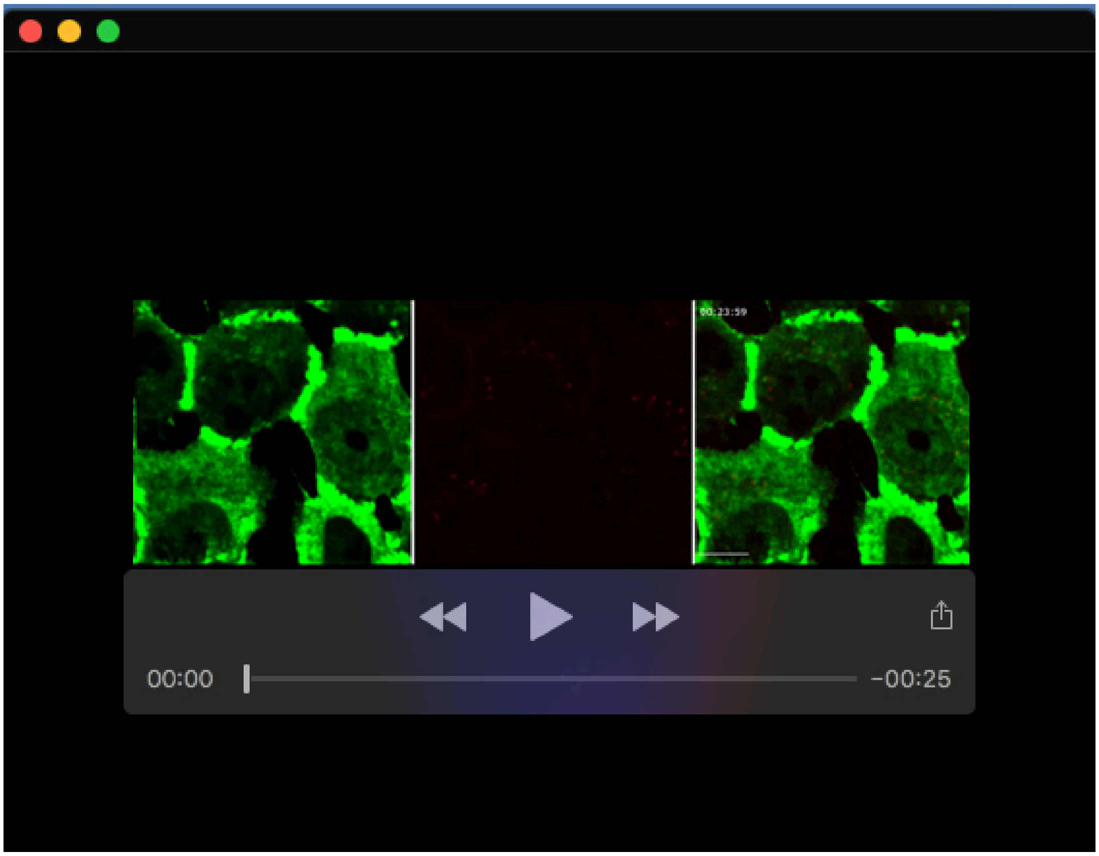

Movie 2. mNG-Rac1 cells were serum starved, placed onto microscope stage into environmental chamber, and 3D live-cell images were acquired through 488-nm (*green*) and 561-nm (*red*) channels from living cells at 37°C for 60 min. EGF-Rh (final concentration 4 ng/ml) was perfused 5 min after the start of time-lapse imaging. Images were deconvolved. Imaging parameters for acquiring EGF-Rh images were used to avoid saturating signals at later time points, which resulted in the lack of visible EGF-Rh fluorescence at early time points. MIP images of 4 consecutive confocal planes are presented. Scale bar, 10 μm.
