# Supplementary material for: Cell migration signaling through the EGFR-VAV2-Rac1 pathway is sustained in endosomes
Source: J Cell Sci. 2025 Jan 24;138(2):jcs263541. doi: 10.1242/jcs.263541 (PMC11828472; doi:10.1242/jcs.263541)
Supplement: Supplementary file 1 [file joces-138-263541_review_history.pdf]

Journal of Cell Science

Cell migration signaling through the EGFR-VAV2-Rac1 pathway is sustained in endosomes

Itziar Pinilla-Macua, Sachin Surve and Alexander Sorkin

DOI: 10.1242/jcs.263541

Editor: Daniel Billadeau

Review timeline

Original submission:	13 September 2024
Editorial decision:	10 October 2024
First revision received:	15 December 2024
Accepted:	16 December 2024

Original submission

First decision letter

MS ID#: JOCES/2024/263541

MS TITLE: Cell migration signaling through EGFR-VAV2-Rac1 pathway is sustained in endosomes

AUTHORS: Itziar Pinilla-Macua, Sachin Surve and Alexander Sorkin

We have now reached a decision on the above manuscript.

To see the reviewers' reports and a copy of this decision letter, please go to:

As you will see, the reviewers gave favourable reports but raised some minor points that will require amendments to your manuscript. I hope that you will be able to carry these out because I would like to be able to accept your paper, depending on further comments from reviewers, if needed.

Reviewer 1

Advance summary and potential significance to field

The study corroborate and reinforce previous studies showing that EGF binding to EGFR triggers cell motility through the EGFR-VAV2-Rac1 pathway, which continues to signal even after EGFR is internalized via clathrin-mediated endocytosis when cell are stimulated with close to physiological doses of ligands. Using live-cell microscopy on human oral squamous carcinoma HSC3 cells, the authors show that VAV2, a guanine nucleotide exchange factor, co-endocytoses with EGFR and is necessary for chemotactic migration and to sustain RAC1 activation. This work suggests that EGFR-dependent cell migration relies on ongoing signaling from endosomes, providing confirmatory experimental evidence of previous publsihed results into the role of EGFR endocytosis in cancer cell motility.

Comments for the author

This is a straightforward study exploring the role of endosomal signaling in regulating EGF-dependent guided migration. While conceptually not highly novel, the work presents a rigorous set

© 2024. Published by The Company of Biologists under the terms of the Creative Commons Attribution License (https://creativecommons.org/licenses/by/4.0/).

of experiments supporting the role of VAV2-RAC1 endosomal signaling in controlling EGF-guided migration. However, several questions and points of curiosity should be addressed:

1. The use of the HSC3 cell line, which depends on EGF, is appropriate. The authors stimulate these cells with 4 ng/ml of EGF, which they argue is close to physiological levels. However, higher concentrations of EGF can occur in certain contexts. Additionally, stimulation with saturating doses of EGF (>10 ng/ml) has been shown to activate both clathrin-dependent and non-clathrin-dependent pathways. The latter promotes massive EGFR internalization into endosomes and eventually into lysosomes, which has been shown to enhance cell migration in some cell types. It would be relevant to test the distribution of VAV2 and RAC1 after high doses of EGF and assess how VAV2 loss-of-function affects migration in response to higher EGF doses.

2. The authors examine the impact of VAV2 loss-of-function on chemotactic migration. It would be informative to also assess whether chemokinesis is affected, and whether migration toward different chemotactic signals, such as serum or LPA, is altered. Is VAV2 specifically required for EGF chemotaxis, or does its loss affect migration toward other stimuli? The authors discuss VAV2's role in HGF-mediated responses"does migration toward HGF get equally disrupted by VAV2 loss? Is random motility affected by loss of VAV2?

3. On page 8, line 14, the reference to Thalappilly et al. focuses on VAV2's role in EGFR endocytosis. More relevant references from the Di Fiore and Sigismund groups, which discuss non-clathrin EGFR internalization pathways, should also be cited.

4. In discussing the results of Figure 5B, the authors state, "These experiments demonstrate that VAV2-mediated EGF-guided cell migration requires CME." However, the experiment primarily shows that clathrin heavy chain (CHC) is required for optimal migration toward EGF. It does not definitively prove that CHC is specifically required for VAV2-mediated migration. This conclusion should be toned down for accuracy.

5. The discussion suggests that TIAM may not be involved in EGFR-guided migration in HSC3 cells. To support this claim, a simple siRNA experiment targeting TIAM could be conducted.

Overall, while the manuscript presents a solid body of work, addressing these points would strengthen and extend the conclusions and provide additional insights into VAV2's role in EGF-guided migration

<u>Reviewer 2</u>

Advance summary and potential significance to field

This study deals with the role of the EGFR-VAV2-Rac1 signaling pathway in cell migration, with a particular focus on endosomal signaling. The authors demonstrate that ligand binding to EGFR activates VAV2, which is subsequently endocytosed with EGFR into endosomes. This signaling, sustained within endosomes, is crucial for the activation of Rac1, leading to downstream actin remodeling.

Their finding of the EGFR-VAV2-Rac1 signaling axis driving cell motility is a significant contribution to the field of cell biology. I believe that this work potentially holds broad relevance for cancer biology because of the cell line and pathway studied. The experiments are well designed, and the authors' interpretations of the results are sound. Their claims are largely fair and well supported by the data, with the exception of one overstatement, which I have noted below. While I do not believe additional experimental data are necessary, I recommend the authors revise some of the presentations as highlighted in my comments.

Comments for the author

1. Control: For the experiments shown in Figures 2A and 7, the authors should either include or describe a control condition in which only simple mNG is expressed in the cells.

© 2024. Published by The Company of Biologists under the terms of the Creative Commons Attribution License (https://creativecommons.org/licenses/by/4.0/).

2. Figure 2A: The authors mention autofluorescence in the main text as well as in the figure legend, but no corresponding data is shown. I recommend that the authors include autofluorescence images in Figure 2A as a reference for readers.

3. Figure 2B: The authors should specify the number of independent experiments used to generate the data. If the dataset consists of results from multiple runs, it would be helpful for readers if the data points from each run were distinguished, for example, by using different colors.

4. Page 7, 1st paragraph: Please ensure that specific figure panels are referenced instead of simply stating "Fig. 3." For example, I believe the reference to Fig. 3 on line 6 should be Fig. 3A, and the one on lines 11/12 should be Fig. 3B.

5. Page 7, line 12: "A significant number of sorting endosomes (containing both transferrin-Alexa647 and LysoTrackerBlue) were positive for EGF-Rh/mNG-VAV2 during the first hour of EGF-Rh stimulation (Fig. 3)." It requires quantification to substantiate this claim.

6. Figure 4B: There is a notable discrepancy between the number of migrated cells in the VAV2KO condition without EGF across the two graphs, despite the small variance within each graph. What explains this difference? Additionally, comparing the Parental control and VAV2 KO conditions without EGF in the left graph, it appears that VAV2 KO increases the number of migrated cells, which suggests that VAV2 KO enhances migration in the absence of EGF. This seems to contradict the statement in the main text that "Elimination of VAV2 significantly reduced the number of cells migrated towards the EGF gradient but did not affect EGF-independent migration (Fig. 4B)" (p7, line 52). The authors should clarify this point.

7. Page 8: The statement "Together, the data in Figure 4 demonstrate that HSC3 cells are a valid model to study the role of endocytosis in the EGFR-VAV2 signaling axis which promotes actin cytoskeleton rearrangement and cell motility" is an overstatement, as the authors do not examine actin cytoskeleton rearrangement. This description should be toned down.

8. Videos: To further enhance the readers' understanding, consider adding videos that represent the dynamics of cell responses, particularly for Figures 2 and 7.

First revision

Response to Reviewers

Dear Dr. Billadeau:

We would like to submit a revised version of the manuscript, "Cell migration signaling through the EGFR-VAV2-Rac1 pathway is sustained in endosomes," for consideration in the *Journal of Cell Science*. We greatly appreciate the reviewers' favorable evaluation of our work. Although their comments were editorially categorized as "minor," addressing them required extensive additional experimentation. Consequently, we have included six new supplemental figures, two videos, and modified or added five new panels in the main figures. Changes to the main text are indicated in red. Our detailed responses to all reviewers' comments are outlined in the accompanying rebuttal.

Most revisions are straightforward, encompassing additional controls and quantitative analyses of the data. However, we wish to highlight our response to Reviewer 1's comments regarding high EGF concentrations and clathrin-independent endocytosis. As discussed in the rebuttal and on page 5 of the revised manuscript, we have not found evidence supporting the existence of "high" EGFR ligand concentrations in tumors *in vivo* using single-cell or biochemical approaches (eLife, 2017). Furthermore, to our knowledge, clathrin-independent endocytosis of EGFR has not been demonstrated experimentally *in vivo*. For these reasons, we have focused in recent years on the mechanisms and functional role of clathrin-mediated endocytosis of EGFR. While high concentrations of EGFR ligands are technically simpler to use, we perform our experiments with low

© 2024. Published by The Company of Biologists under the terms of the Creative Commons Attribution License (https://creativecommons.org/licenses/by/4.0/).

concentrations whenever feasible, balancing the need for a sufficient signal-to-noise ratio. Nevertheless, we have included new experiments with high EGF concentrations in this revision, though integrating these data into the manuscript presented challenges.

We believe that these revisions significantly strengthen the manuscript, and we hope the revised version is now suitable for publication in the *Journal of Cell Science*. While we understand the constraints, and that it is likely unrealistic, we would be deeply grateful if a decision could be made before the end of the year.

REBUTTAL

Reviewer 1: The study corroborate and reinforce previous studies showing that EGF binding to EGFR triggers cell motility through the EGFR-VAV2-Rac1 pathway, which continues to signal even after EGFR is internalized via clathrin-mediated endocytosis when cell are stimulated with close to physiological doses of ligands. Using live-cell microscopy on human oral squamous carcinoma HSC3 cells, the authors show that VAV2, a guanine nucleotide exchange factor, co- endocytoses with EGFR and is necessary for chemotactic migration and to sustain RAC1 activation. This work suggests that EGFR-dependent cell migration relies on ongoing signaling from endosomes, providing confirmatory experimental evidence of previous publsihed results into the role of EGFR endocytosis in cancer cell motility.

SUGGESTIONS TO AUTHORS.
This is a straightforward study exploring the role of endosomal signaling in regulating EGF-dependent guided migration. While conceptually not highly novel, the work presents a rigorous set of experiments supporting the role of VAV2-RAC1 endosomal signaling in controlling EGF-guided migration. However, several questions and points of curiosity should be addressed:

We agree with the reviewer that our findings conceptually align with studies demonstrating the role of clathrin-mediated endocytosis in HGF/MET-VAV2-Rac1 signaling to cell migration. However, we have been unable to identify publications that experimentally demonstrate the requirement of EGFR endocytosis for cancer cell motility, whether involving the VAV2-RAC1 axis or other signaling pathways. While it is possible we missed relevant studies among the over 120,000 PubMed entries for the keyword “EGFR,” many studies report the opposite—namely, that cell migration is enhanced by retaining EGFR at the cell surface. Moreover, based on our experience re-evaluating the role of endosomes in the Ras-MAPK pathway using live-cell imaging of endogenous proteins, we recognize the importance of conducting similar studies in other signaling systems, including the RTK-VAV2-Rac1 axis.

1. The use of the HSC3 cell line, which depends on EGF, is appropriate. The authors stimulate these cells with 4 ng/ml of EGF, which they argue is close to physiological levels. However, higher concentrations of EGF can occur in certain contexts. Additionally, stimulation with saturating doses of EGF (>10 ng/ml) has been shown to activate both clathrin-dependent and non-clathrin-dependent pathways. The latter promotes massive EGFR internalization into endosomes and eventually into lysosomes, which has been shown to enhance cell migration in some cell types. It would be relevant to test the distribution of VAV2 and RAC1 after high doses of EGF and assess how VAV2 loss-of-function affects migration in response to higher EGF doses.

Response. Our studies in mouse tumor xenografts of HSC3 and two other EGFR-dependent cancer cells demonstrated that EGFR ligands are present in picomolar concentrations (<1-2 ng/ml) in vivo and that these concentrations are sufficient for sustaining tumor progression (Pinilla-Macua et al., eLife 2017). In these studies, we have not found any evidence for the presence of high (>10 ng/ml) EGFR ligand concentrations and clathrin-independent endocytosis of the receptor *in vivo* using biochemical and single-cell microscopy analyses (Pinilla-Macua, eLife 2017). Please see page 5, bottom paragraph.

© 2024. Published by The Company of Biologists under the terms of the Creative Commons Attribution License (https://creativecommons.org/licenses/by/4.0/).

Therefore, we perform our experiments with low EGF concentrations whenever feasible (significant signal-to-noise ratio in an outcome of an assay). Nonetheless, as reviewer requested, we performed new experiments to show colocalization of EGF-Rh used in high concentration (40 ng/ml; subsaturating concentration for HSC3 cells expressing ~500,000 EGFR/cell) with mNG-VAV2 and mNG-Rac1 cells. Please see new Figures S2 and page 6, bottom; Figure S7 and page 12, top paragraph.

We have also performed chemotactic cell migration experiments using 40 ng/ml EGF. Surprisingly, this EGF concentration did not promote cell migration (Figure S3), presumably because of EGF-induced EGFR down-regulation under these experimental conditions. Please see page 8, lines 15-23 for description of these experiments.

2. The authors examine the impact of VAV2 loss-of-function on chemotactic migration. It would be informative to also assess whether chemokinesis is affected, and whether migration toward different chemotactic signals, such as serum or LPA, is altered. Is VAV2 specifically required for EGF chemotaxis, or does its loss affect migration toward other stimuli? The authors discuss VAV2's role in HGF-mediated responses—does migration toward HGF get equally disrupted by VAV2 loss? Is random motility affected by loss of VAV2?

Response. As reviewer suggested, we performed experiments to show that HSC3 cells are not chemoattracted to FBS but migrate towards the HGF gradient in a VAV2-dependent manner. With regards to the effects on random migration, we focused in this study on previously documented effects of VAV2 on cell motility, such as chemotactic migration and substrate spreading. We believe that a more comprehensive characterization of the role of VAV2 in various types of cell motility is the subject of future studies, which we hope will be facilitated by the availability of our newly generated VAV2KO cells. We have analyzed the role of VAV2 in the wound-healing assay, but the results were inconsistent with no statistical significance to support the role of VAV2 in this random cell motility.

3. On page 8, line 14, the reference to Thalappilly et al. focuses on VAV2's role in EGFR endocytosis. More relevant references from the Di Fiore and Sigismund groups, which discuss non-clathrin EGFR internalization pathways, should also be cited.

Response. We have not been able to find PubMed entrees for keywords "[VAV2] and [EGFR]", and authored by Drs. Di Fiore or Sigismund. We apologize if we still missed such studies. If the reviewer meant that we cite papers demonstrating clathrin-independent endocytosis in HeLa cells used by Thalappilly et al., we modified the text and included the original citation on clathrin- independent endocytosis (Sigismund et al., 2005) on page 8, bottom line.

4. In discussing the results of Figure 5B, the authors state, "These experiments demonstrate that VAV2-mediated EGF-guided cell migration requires CME." However, the experiment primarily shows that clathrin heavy chain (CHC) is required for optimal migration toward EGF. It does not definitively prove that CHC is specifically required for VAV2-mediated migration. This conclusion should be toned down for accuracy.

Response. This is corrected. Please see page 9, 3rd paragraph.

5. The discussion suggests that TIAM may not be involved in EGFR-guided migration in HSC3 cells. To support this claim, a simple siRNA experiment targeting TIAM could be conducted.

Response. Unfortunately, we were unable to detect TIAM1 in HSC3 cells using published antibodies (Menard et al., 2014), suggesting a low concentration of TIAM1 in these cells. However, we have conducted a new series of experiments demonstrating that EGF-induced Rac1 activation in HSC3 cells is highly dependent on VAV2 (Figures 6D-F and S5). Given this, we believe that investigating the role of TIAM in Rac1 activation and EGFR-mediated cell migration falls outside the scope of this

© 2024. Published by The Company of Biologists under the terms of the Creative Commons Attribution License (https://creativecommons.org/licenses/by/4.0/).

manuscript, which focuses specifically on VAV2. Such a study would require a substantial independent effort, including antibody screening and gene- editing approaches, which would be both costly and time-consuming. Therefore, we minimized the discussion about TIAM by omitting the sentence from page 12, second paragraph: “Furthermore, TIAM-1 was not found in the proximity proteome of internalized EGFR in HCC116 and HEK293 cells whereas Rab5 and VAV2 were enriched in this proximity proteome (Perez Verdaguer et al., 2022).”

Reviewer 2:

1. Control: For the experiments shown in Figures 2A and 7, the authors should either include or describe a control condition in which only simple mNG is expressed in the cells.

Response. We described the difference in the localization of unfused “simple” mNG and mNG-edited proteins in the “Methods” section. Please see page 15, lines 11-17.

2. Figure 2A: The authors mention autofluorescence in the main text as well as in the figure legend, but no corresponding data is shown. I recommend that the authors include autofluorescence images in Figure 2A as a reference for readers.

Response. We have now included the image of non-specific fluorescence (640 nm channel) for the 20-min timepoint presented in Figure 2 (please see Figure S1A). We have also presented images of mNG-VAV2 and parental cells acquired through 488 nm channel using identical parameters, and quantification of these images in Figure S1B-C to directly demonstrate the specificity of mNG-VAV2 fluorescence in endosomes.

3. Figure 2B: The authors should specify the number of independent experiments used to generate the data. If the dataset consists of results from multiple runs, it would be helpful for readers if the data points from each run were distinguished, for example, by using different colors.

Response. The graph was revised to show that it is the result of 3 independent experiments by differently labeled symbols.

4. Page 7, 1st paragraph: Please ensure that specific figure panels are referenced instead of simply stating "Fig. 3." For example, I believe the reference to Fig. 3 on line 6 should be Fig. 3A, and the one on lines 11/12 should be Fig. 3B.

Response. Apologies for our sloppiness. This is corrected.

5. Page 7, line 12: "A significant number of sorting endosomes (containing both transferrin-Alexa647 and LysoTrackerBlue) were positive for EGF-Rh/mNG-VAV2 during the first hour of EGF-Rh stimulation (Fig. 3)." It requires quantification to substantiate this claim.

Response. These data are now quantitated. Please see Figure 3C and corresponding section in the “Methods” section (page 18).

6. Figure 4B: There is a notable discrepancy between the number of migrated cells in the VAV2KO condition without EGF across the two graphs, despite the small variance within each graph. What explains this difference? Additionally, comparing the Parental control and VAV2 KO conditions without EGF in the left graph, it appears that VAV2 KO increases the number of migrated cells, which suggests that VAV2 KO enhances migration in the absence of EGF. This seems to contradict the statement in the main text that "Elimination of VAV2 significantly reduced the number of cells migrated towards the EGF gradient but did not affect EGF- independent migration (Fig. 4B)" (p7, line 52). The authors should clarify this point.

© 2024. Published by The Company of Biologists under the terms of the Creative Commons Attribution License (https://creativecommons.org/licenses/by/4.0/).

Response. Figure 4B includes examples of individual experiments, each with triplicate inserts. We apologize for not clearly describing this panel in the legend. Because of some variability in cell counts in this type of cell migration assays, we have now presented a combined analysis of 3 independent experiments, exemplified in Figure 4B, in new Figure 4C. Statistical analysis revealed no significant differences in the migration of parental, mNG-VAV2 and VAV2KO cells in the absence of EGF.

7. Page 8: The statement "Together, the data in Figure 4 demonstrate that HSC3 cells are a valid model to study the role of endocytosis in the EGFR-VAV2 signaling axis which promotes actin cytoskeleton rearrangement and cell motility" is an overstatement, as the authors do not examine actin cytoskeleton rearrangement. This description should be toned down.

Response. This is corrected by omitting “actin rearrangement” (page 9, end of top paragraph).

8. Videos: To further enhance the readers' understanding, consider adding videos that represent the dynamics of cell responses, particularly for Figures 2 and 7.

Response. We have now added videos supplemental to Figures 2 and 7 (Movies 1 and 2).

Reviewer 3:
There are some minor comments that the authors may wish to consider:

1) By its SH2 domain, Vav2 is expected to bind directly to phosphorylated EGFR, as indicated in the manuscript on page 4, lines 34-39. Several studies have examined how EGFR phosphorylation may be regulated by clathrin endocytosis, largely finding that EGF-stimulated EGFR phosphorylation does not require internalization. It may nonetheless be useful to include either a citation of a study that previously examined the impact of clathrin perturbation on EGFR pY992 and pY1178 (the sites of Vav2 binding reported by Tamas et al 2003) or to probe this in the HSC3 cells examined here. This would help define whether to expect the selective recruitment of Vav2 to endosomes (likely via interaction with phosphorylated EGFR) to be a reflection of selective EGFR phosphorylation at these sites, or more likely the consequence of more complex signaling regulation downstream of the receptor.

Response. We now demonstrate in new experiments that phosphorylation of EGFR at Tyr1173 and Tyr992 is sustained in CHC-depleted cells. Please see Figure S4, page 9, end of second paragraph.

2) It may be worthwhile to determine if Rac1 GTP loading upon EGF stimulation is Vav2 dependent in the HSC3 cell model. The manuscript describes that activation of Rac1 likely has cell-type specific activation requirements in terms of GEFs, so this would be useful information to support the model presented. A role for Vav2 could be shown by perturbation of Vav2 followed by detection of Rac1 localization or Rac1 GTP loading determined by pull-down.

Response. Thank you for this suggestion. This is indeed an important experiment which we should have included in the original submission. We have performed GTP-Rac1 pulldown experiments in mNG-VAV2 and VAV2KO cells (these cells were derived from mNG-VAV2 cells). These experiments demonstrated that EGF activation of Rac1 is highly dependent on VAV2. Please see new Figures 6D-E. In addition, we confirmed this conclusion by RNAi knockdown of VAV2. Please see the new Figure S5.

3) In reference to the Results section, page 6, lines 19-21 and Figure 2B, were the FOVs subject to quantification as shown in Figure 2B from a single or multiple independent experiments? This information should be noted in the figure legend of Figure 2B. If these measurements were not from independent experiments, it might be worth tempering the conclusion of the specific percentage of mNG-Vav2 localizing to endosomes. This is a minor point that does not take away

© 2024. Published by The Company of Biologists under the terms of the Creative Commons Attribution License (https://creativecommons.org/licenses/by/4.0/).

from the strength of the overall conclusion that EGF stimulation triggers recruitment of Vav2 to endosomes, which is quite evident from the images and data presented.

Response. The graph was revised to show that it is the result of 3 independent experiments by differently labeled symbols.

4) In the Results section, page 7, lines 52-27, the effect of Vav2 KO is described as being specific to the EGF-stimulated condition, as this "did not affect EGF-independent migration" (referencing Figure 4B). It seems that in control (par) cells, in the absence of EGF stimulation, there is no detectable cell migration. It appears that in Vav2 KO cells, this migration in the absence of EGF stimulation is perhaps increased, but this is not statistically compared. It is difficult to make conclusions about changes to EGF-independent migration in this transwell assay since there is no or little detectable transwell migration in the parental cells in the absence of EGF stimulation. Some tempering of this statement may be warranted.

Response. Figure 4B includes examples of individual experiments, each with triplicate inserts. We apologize for not clearly describing this panel in the legend. Because of some variability in cell counts in this type of cell migration assays, we have now presented a summary of the analysis of 3 independent experiments, exemplified in Figure 4B, in new Figure 4C. Statistical analysis revealed no significant differences in the migration of parental, mNG-VAV2 and VAV2KO cells in the absence of EGF.

<u>Second decision letter</u>

MS ID#: JOCES/2024/263541

MS TITLE: Cell migration signaling through EGFR-VAV2-Rac1 pathway is sustained in endosomes

AUTHORS: Itziar Pinilla-Macua, Sachin Surve and Alexander Sorkin

I am happy to tell you that your manuscript has been accepted for publication in Journal of Cell Science, pending standard publication integrity checks.

© 2024. Published by The Company of Biologists under the terms of the Creative Commons Attribution License (https://creativecommons.org/licenses/by/4.0/).
